# Effectiveness of an Intervention Supporting Shared Decision Making for Destination Therapy Left Ventricular Assist Device: The DECIDE-LVAD Randomized Clinical Trial

**DOI:** 10.1001/jamainternmed.2017.8713

**Published:** 2018-02-26

**Authors:** Larry A. Allen, Colleen K. McIlvennan, Jocelyn S. Thompson, Shannon M. Dunlay, Shane J. LaRue, Eldrin F. Lewis, Chetan B. Patel, Laura Blue, Diane L. Fairclough, Erin C. Leister, Russell E. Glasgow, Joseph C. Cleveland, Clifford Phillips, Vicie Baldridge, Mary Norine Walsh, Daniel D. Matlock

**Affiliations:** 1 Adult and Child Consortium for Outcomes Research and Delivery Science, University of Colorado School of Medicine, Aurora; 2 Division of Cardiology, University of Colorado School of Medicine, Aurora; 3 Department of Cardiovascular Diseases, Mayo Clinic, Rochester, Minnesota; 4 Washington University School of Medicine, St Louis, Missouri; 5 Brigham and Women's Hospital, Boston, Massachusetts; 6 Duke University Medical Center, Durham, North Carolina; 7 Department of Biostatistics and Informatics, University of Colorado School of Public Health, Aurora; 8 Veterans Affairs, Eastern Colorado Geriatric Research Education and Clinical Center, Denver; 9 Division of Cardiothoracic Surgery, University of Colorado School of Medicine, Aurora; 10 Patient stakeholder; 11 Caregiver stakeholder; 12 Division of Cardiology, St Vincent Heart Center, Indianapolis, Indiana; 13 Division of Geriatric Medicine, University of Colorado School of Medicine, Aurora

## Abstract

**Importance:**

Shared decision making helps patients and clinicians elect therapies aligned with patients’ values and preferences. This is particularly important for invasive therapies with considerable trade-offs.

**Objective:**

To assess the effectiveness of a shared decision support intervention for patients considering destination therapy left ventricular assist device (DT LVAD) placement.

**Design, Setting, and Participants:**

From 2015 to 2017, a randomized, stepped-wedge trial was conducted in 6 US LVAD implanting centers including 248 patients being considered for DT LVAD. After randomly varying time in usual care, sites were transitioned to an intervention consisting of clinician education and use of DT LVAD pamphlet and video patient decision aids. Follow up occurred at 1 and 6 months.

**Main Outcomes and Measures:**

Decision quality as measured by knowledge and values-choice concordance.

**Results:**

In total, 135 patients were enrolled during control and 113 during intervention periods. At enrollment, 59 (23.8%) participants were in intensive care, 60 (24.1%) were older than 70 years, 39 (15.7%) were women, 45 (18.1%) were racial/ethnic minorities, and 62 (25.0%) were college graduates. Patient knowledge (mean test performance) during the decision-making period improved from 59.5% to 64.9% in the control group vs 59.1% to 70.0% in the intervention group (adjusted difference of difference, 5.5%; *P* = .03). Stated values at 1 month (scale 1 = “do everything I can to live longer…” to 10 = “live with whatever time I have left…”) were a mean of 2.37 in control and 3.33 in intervention (*P* = .03). Patient-reported treatment choice at 1 month favored LVAD more in the control group (than in the intervention group (47 [59.5%] vs 95 [91.3%], *P* < .001). Correlation between stated values and patient-reported treatment choice at 1 month was stronger in the intervention group than in the control group (difference in Kendall’s τ, 0.28; 95% CI, 0.05-0.45); however, there was no improved correlation between stated values and actual treatment received by 6 months for the intervention compared with the control group (difference in Kendall’s τ, 0.01; 95% CI, −0.24 to 0.25). The adjusted rate of LVAD implantation by 6 months was higher for those in the control group (79.9%) than those in the intervention group (53.9%, *P* = .008), with significant variation by site. There were no differences in decision conflict, decision regret, or preferred control.

**Conclusions and Relevance:**

A shared decision-making intervention for DT LVAD modestly improved patient decision quality as measured by patient knowledge and concordance between stated values and patient-reported treatment choice, but did not improve concordance between stated values and actual treatment received. The rate of implantation of LVADs was substantially lower in the intervention compared with the control group.

**Trial Registration:**

clinicaltrials.gov Identifier: NCT02344576

## Key Points

**Question:**

Does a shared decision support intervention for patients considering destination therapy left ventricular assist device (DT LVAD) improve decision quality compared with usual care?

**Findings:**

In this multicenter randomized stepped-wedgeclinical trial of 248 patients being considered for DT LVAD, compared with patients in the usual care control period, patients enrolled in the intervention period had significantly better knowledge and higher concordance between stated values and patient-reported treatment choice.

**Meaning:**

An intervention supporting shared decision making for DT LVAD was associated with improved patient decision quality.

## Teaser

This randomized clinical trial examines the effectiveness of a shared decision support intervention for patients considering destination therapy left ventricular assist device placement.

## Introduction

Shared decision making is a process that helps patients and clinicians align therapies with patients’ values and preferences.^1^ This is particularly important for invasive therapies for life-threatening illness,^2^ such as left ventricular assist devices as destination therapy (DT LVADs). These devices are offered to patients with severe heart failure who are ineligible for cardiac transplantation. Placement of a DT LVAD can be life-prolonging, but also comes with considerable changes in lifestyle, need for caregiver support, and a high likelihood of complications.^4^ Unfortunately, for many major decisions involving newer medical technologies—including DT LVAD—education, consent, and shared decision-making processes are suboptimal.^6^ Current consent documents are too long and poorly written,^8^ industry materials tend to be biased,^9^ and clinicians often lack the skills to support high-quality decision making.^11^

Patient decision aids are a form of decision support that standardize the information received by patients to support a shared decision-making process.^12^ Decision aids have been shown to improve knowledge and reduce decisional conflict.^13^ However, few tools have been developed to engage seriously ill patients in shared decision making,^14^ and until recently, none were available for LVAD.^9^ In addition, the effectiveness of decision aids in clinical practice is largely unknown and wide-scale implementation remains a substantial challenge.^15^

In this context, we performed a series of studies exploring the decisional needs for patients and their caregivers considering DT LVAD.^10^ Based on this research and following the International Patient Decision Aid Standards (IPDAS), we developed pamphlet and video decision aids for patients and their caregivers considering DT LVAD.^19^ We aimed to study the effectiveness of these decision aids coupled with a clinician-directed support training through a multicenter, cluster-randomized, stepped-wedge design.

## Methods

### Design and Sites

The Multicenter Trial of a Shared Decision Support Intervention for Patients and their Caregivers Offered Destination Therapy for End-Stage Heart Failure (DECIDE-LVAD) used a hospital-level, randomized phased roll out (stepped wedge) in 6 mechanical circulatory support (MCS) programs across the United States.^20^ This approach was chosen because the intervention engages clinicians and other program staff in addition to patients.^22^ The study was overseen by the institutional review board at the University of Colorado and approved by institutional review boards at all sites. The trial protocol is available in Supplement 1.

### Study Participants

Patient and caregiver dyads were enrolled from the 6 sites during a 20-month enrollment period. Patient eligibility criteria included age 18 years or older, end-stage heart failure, and active consideration for a DT LVAD. Eligible patients were identified by the study team at each site at the time a DT LVAD evaluation was initiated. This was triggered either by a preauthorization request to the patient’s health insurance for LVAD evaluation or a provider’s request for formal education about LVAD. Written informed consent was obtained from all study participants. Participants were compensated $25 for completing surveys at each time point.

### Intervention

The content and development of the decision aids is described separately^19^; the pamphlet is available in Supplement 2**.**

All sites began in the control period using their existing materials during formal education. This process typically consisted of a patient teaching session with an MCS coordinator and use of industry pamphlets/videos and program-specific documents. At 4 stepped time intervals, programs were randomly assigned to cross over to the intervention. The decision support intervention included (1) delivery of a 2.5-hour clinician-directed decision support training and (2) use of the 26-minute video and 8-page pamphlet decision aids developed by our group.^19^ At the time of intervention implementation, staff directly involved in LVAD patient education and care were asked to attend a 60-minute grand rounds style presentation about shared decision making for DT LVAD, followed by a 90-minute coaching session that included demonstration and discussion of the decision aid materials.^20^ With facilitation by local physician champions, sites were then instructed to formally integrate the decision aids and tenets learned from the coaching session into existing education, decision making, and informed consent processes. The only requirements of sites around the use of the decision aids were that they be delivered by clinicians and not research staff. This design allowed for sites to implement the decision aids in a way that was most appropriate for that site; thus, local differences in how the intervention was delivered were possible.

### Data Collection

Data collection was the same during both control and intervention periods. For all patients meeting initial eligibility criteria, basic demographic and health status data were captured in the screening form. Enrolled participants were administered surveys (Supplement 2) at 4 time points: prior to formal LVAD education (baseline 1), immediately after formal education (baseline 2), 1 month after enrollment, and 6 months after enrollment. Baseline surveys were administered in person (with verbal assessments completed),^23^ whereas follow-up surveys were administered in person or through telephone or mail. Medical records were reviewed at enrollment, 1 month, and 6 months for relevant clinical information, outcomes, and adverse events. Decision aid use by individual patients was reported by clinical staff. All data were entered into a REDCap database by the individual sites.^26^

### Outcomes

The primary outcome was decision quality: the extent to which medical decision making reflects the considered preferences of a well-informed patient.^13^ As such, coprimary endpoints were chosen comprising the 2 main IPDAS domains of decision quality—knowledge and values-choice concordance.

A 10-item knowledge test was developed by the study team and validated by clinicians and patients. Consistent with the methods of Sepucha et al,^29^ the study team created a list of knowledge items based on clinical needs, local post-LVAD education standards, and needs assessment work with patients. We then surveyed patients, caregivers, MCS coordinators, and physicians to narrow the list and determine the key knowledge items and assure content validity. The acceptability of this measure was further assessed with patients and caregivers in a pilot of the trial protocol.^20^ Improvement in knowledge from baseline 1 to baseline 2 was a coprimary endpoint.^30^ A values scale was also developed, modeled after a well-accepted values evaluation tool.^31^ During previous needs assessment work,^10^ 1 value rose above all others in considering DT LVAD: maximizing chances of survival with aggressive medical care vs not. We developed a single-item, 10-tier Likert values measure using the dichotomy of “Do everything I can to live longer, even if that means having major surgery and being dependent on a machine” (score 1) vs “Live with whatever time I have left, without going through major surgery or being dependent on a machine” (score 10). Concordance between 1-month value score and patient-reported treatment choice (DT LVAD or medical treatment without LVAD) at 1 month was the other coprimary endpoint. Concordance between 1-month value score and actual treatment received by 6 months was also assessed.

Secondary outcomes included validated measures of decision conflict,^32^ decision regret,^33^ control preferences,^34^ illness acceptance,^35^ perceived stress,^36^ depression (Patient Health Questionnaire-2),^37^ and quality of life (EuroQol Visual Analogue Scale).^38^ Acceptability of the decision aids was also measured at baseline 2.^39^

### Analysis

We determined that a sample size of 168 participants with standard deviation of 18% would yield a power of 0.95 to detect an improvement in knowledge by 10%. We anticipated a dropout rate of 25% by 6 months based on expected death rates and other loss to follow-up.

We compared baseline characteristics between participants enrolled in the study to those screened but not enrolled using χ^2^ tests. We compared characteristics between those enrolled during the control period with those enrolled during the intervention period using χ^2^ tests and *t* tests.

To assess the change in patient DT LVAD knowledge over time, we fit a linear mixed model proposed for the analysis of stepped wedge designs.^40^ This model accounted for the repeated within-person measures, included a random effect for site and fixed effect indicators of intervention group and stepped wedge time period. This model adjusts for trends over time, assuming that changes occur similarly across all sites. Owing to differences observed at baseline, we included 2 covariate indicator variables: outpatient status and diagnosis of heart failure less than 4 years prior to enrollment. We evaluated whether the change in knowledge score (percent correct) between baseline 1 and baseline 2 was different between the control and intervention groups.

To assess values-choice concordance, we calculated the Kendall’s τ correlation coefficient between the stated values score at 1 month and each of the treatment outcomes (patient-reported treatment choice at 1 month, actual treatment received at 6 months), and looked at the difference in this correlation coefficient by intervention group. To generate a confidence interval for this difference, we performed 500 bootstrap samples and calculated the 2.5th and 97.5th percentiles.

In analysis of secondary survey scores described previously and the LVAD implantation rates by intervention group, we applied the same mixed model methods described above. Owing to site differences in LVAD implantation rate and differences in location of patient enrollment over time, we performed separate sensitivity analyses accounting for each, as well as sensitivity analysis without including the site random effect. Missing data analyses can be viewed in Supplement 2.

All analyses were performed using SAS statistical software (version 9.4, SAS Inc).

## Results

### Patients

**Figure 1.**
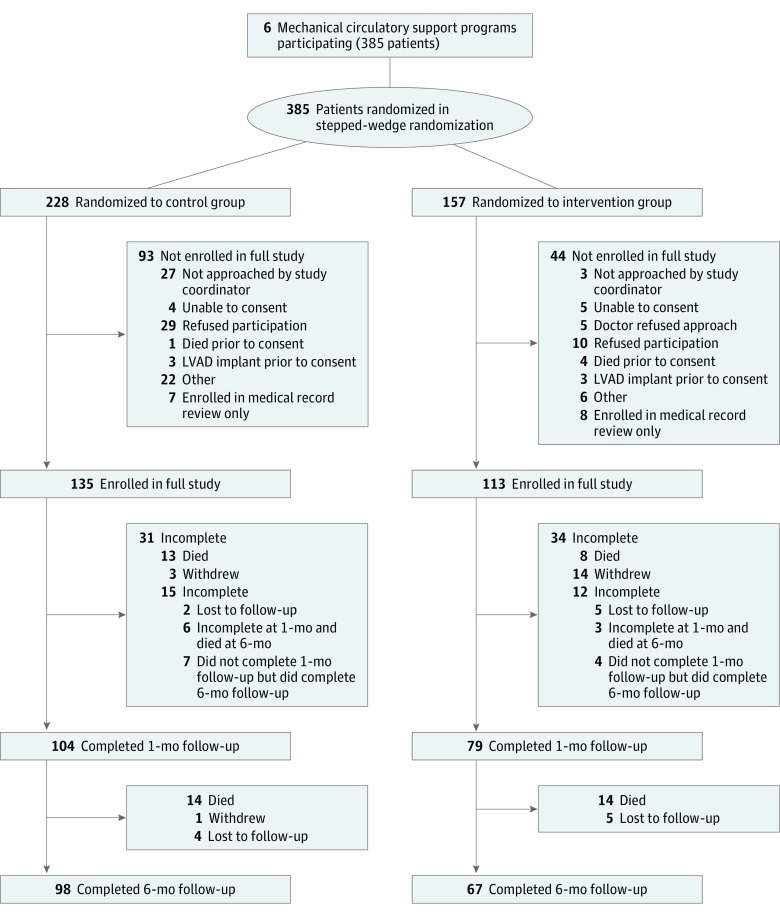
Screening and Enrollment Flow Diagram

Between June 2015 and January 2017, of 385 patients who were actively considering a DT LVAD, 248 were enrolled (Figure 1). Compared with patients who were screened but not enrolled in the study, enrolled patients were more likely to be white non-Hispanic (75.8% vs 63.9%, *P* = .03); other demographics and clinical status were not different between the 2 groups.

Patients in the intervention period were more likely to be enrolled as outpatient (31% vs 17%, *P* = .007) and to have been diagnosed with heart failure less than 4 years prior (29.8% vs 18.2%, *P* = .03) than those enrolled in the control period. See Table 1 for other demographic information.

**Table 1.** Participant Baseline Characteristics

Characteristic	No. (%)	
	Control (n = 135)^a^	Intervention (n = 113)^a^
Age, mean (SD), y^b^	63.5 (9.7)	63.2 (10.2)
Sex, male	111 (82.2)	98 (86.7)
Race/ethnicity^c^		
White, non-Hispanic	102 (79.1)	86 (82.7)
Black	19 (14.7)	12 (11.5)
Other	8 (6.2)	6 (5.8)
Some college or more	74 (56.4)	72 (69.2)
Receiving disability	35 (27.6)	33 (32.0)
Annual household income <$40 000	64 (51.6)	37 (40.2)
Married	95 (72.5)	68 (65.4)
First diagnosed with heart failure		
Within past 2 years	15 (11.9)	12 (12.4)
2-4 years	9 (7.1)	19 (19.6)
4 or more years	102 (81.0)	66 (68.0)
INTERMACS score		
1	5 (4.3)	8 (7.9)
2-3	89 (77.4)	48 (47.5)
4-7	21 (18.3)	45 (44.6)
Comorbidities^d^		
Peripheral vascular disease	7 (5.2)	4 (3.5)
Major stroke	2 (1.5)	0 (0)
Severe diabetes	12 (8.9)	11 (9.7)
Chronic renal disease	31 (23.0)	23 (20.4)
Pulmonary disease	12 (8.9)	4 (3.5)
Liver dysfunction	6 (4.4)	5 (4.4)
History of solid organ or blood cancer	10 (7.4)	8 (7.1)
History of alcohol or illicit drug use	13 (9.6)	12 (10.6)
Enrollment location		
Outpatient	23 (17.0)	35 (31.0)
Inpatient (non-ICU)	83 (61.5)	48 (42.5)
ICU	29 (21.5)	30 (26.5)
Cognitive Function (SPMSQ) Score, mean (SD)^e^	0.7 (1.5)	0.7 (1.2)
Intact functioning	123 (93.9)	94 (93.1)
Mild impairment	5 (3.8)	6 (5.9)
Severe impairment	3 (2.3)	1 (1.0)
Literacy (REALM-R) Score, mean (SD)^f^	6.93 (1.9)	6.95 (2.0)
At risk for poor literacy	30 (23.4)	27 (26.7)
Subjective Numeracy Score, mean (SD)^g^	4.0 (1.1)	4.2 (1.1)

Abbreviations: ICU, intensive care unit; INTERMACS, Interagency Registry for Mechanically Assisted Circulatory Support; REALM-R, rapid estimate of adult literacy in medicine, revised; SPMSQ, short portable mental status questionnaire.

^a^ Some participants refused to answer certain demographic questions; the following items had missing data: race/ethnicity (n = 15), education (n = 13), disability status (n = 18), income (n = 32), marital status (n = 13), heart failure diagnosis timing (n = 25), SPMSQ (n = 16), REALM-R (n = 19), Numeracy Score (n = 14).

^b^ Reported from patient medical record.

^c^ Patient-reported from survey.

^d^ Used from INTERMACS preimplant data collection form, section “Concerns and Contraindications.”

^e^ Number of incorrect answers out of 10 questions.

^f^ Number of correctly read words out of 8 listed.

^g^ Range of 1 to 6, higher numeracy toward 6.

### Exposure to and Impression of Decision Support Materials

In the control period, patients most often received site-specific consent forms, locally made documents, and industry pamphlets/videos during formal education. In the intervention period, 94.7% of patients received the decision aid (99 patients received both pamphlet and video, 2 received pamphlet only, 6 received video only, and 6 received neither); 3 of the 6 sites stopped using industry pamphlets/videos labeled as decision making materials. Patient-reported acceptability of the educational materials was not significantly different between the control and intervention periods.

### Decision Quality: Knowledge and Values-Choice Concordance

**Figure 2.**
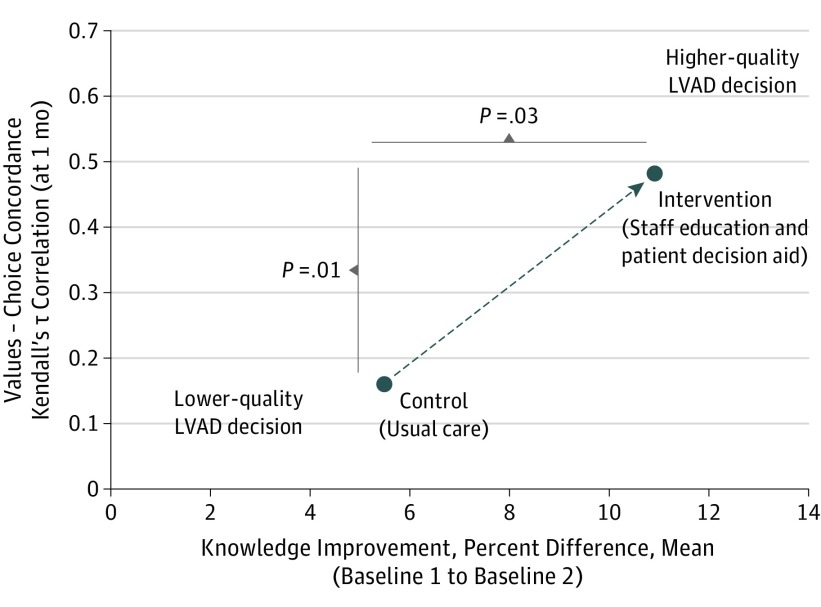
Primary Outcome, Decision Quality LVAD indiciates left ventricular assist device. Decision quality measured by improved knowledge following the education and consent process and concordance between patient values and treatment choice at 1 month after initiation of evaluation for LVAD.

Patient knowledge (mean test performance) during the decision-making period improved from 59.5% to 64.9% in the control group vs 59.1% to 70.0% in the intervention group (difference of difference, 5.5%; *P* = .03) (Figure 2).

Patient values on the 10-tier Likert scale were generally in the direction of more aggressive care to maximize survival (ie, closer to 1): control period baseline 1 score mean of 2.19 (standard error [SE], 0.26); 1-month, 2.37 (SE, 0.28); 6-month, 3.12 (SE, 0.33); intervention period baseline, 2.98 (SE, 0.30); 1-month, 3.33 (SE, 0.32); 6-month, 3.65 (SE, 0.39); adjusted overall difference *P* = .06 (Table 2).

**Table 2.** Outcomes

Outcome	Visit	Control (n = 132)	Intervention (n = 104)	*P* Value
Knowledge score, percent correct (10-item test), mean (SE), %^a^	BL1	59.5 (1.9)	59.1 (2.2)	.92
	BL2	64.9 (1.8)	70.0 (2.1)	.09
	1 mo	67.8 (1.9)	66.4 (2.3)	.64
	6 mo	68.6 (1.8)	67.1 (2.2)	.63
Values score (scale 1-10), mean (SE)^b^	BL1	2.19 (0.26)	2.98 (0.30)	.06
	1 mo	2.37 (0.28)	3.33 (0.32)	.03
	6 mo	3.12 (0.33)	3.65 (0.39)	.32
Treatment choice, “wanted LVAD,” No. (%)^c^	1 mo^d^	95 (92.2)	47 (61.0)	<.001
	6 mo^e^	88 (90.7)	46 (69.7)	.01
Treatment received, LVAD, No. (%)^f^	6 mo	110 (83.3)	54 (52.4)	<.001
HeartMate II^f^	6 mo	68 (61.8)	22 (40.7)	.02
HeartMate 3^f^	6 mo	29 (26.4)	22 (40.7)	.02
HVAD^f^	6 mo	10 (9.1)	10 (18.5)	.02
Decision conflict part b score (0-100), mean (SE)^g^	BL1	20.2 (1.99)	23.4 (2.24)	.28
	BL2	16.5 (1.95)	18.4 (2.23)	.52
	1 mo	15.5 (1.89)	17.9 (2.17)	.42
	6 mo	15.4 (1.89)	14.2 (2.21)	.67
Decision regret score (0-100), mean (SE)^h^	1 mo	14.3 (2.15)	17.9 (2.84)	.37
	6 mo	12.1 (2.28)	19.1 (2.96)	.09
Control preferences scale (preferred), active role, No. (%)^i^	1 mo^d^	90 (86.6)	71 (89.8)	.87
	6 mo^e^	85 (86.7)	61 (92.4)	.70
Control preferences scale (actual), active role, No. (%)^j^	1 mo^d^	91 (87.5)	66 (83.6)	.81
	6 mo^e^	84 (85.8)	59 (89.4)	.95
PEACE: acceptance of illness score (5-20), mean (SE)^k^	BL1	17.5 (0.26)	17.1 (0.31)	.44
	1 mo	17.4 (0.27)	17.4 (0.32)	.90
	6 mo	17.5 (0.28)	18.2 (0.34)	.18
PEACE: struggle with illness score (7-28), mean (SE)^l^	BL1	14.0 (0.42)	13.1 (0.50)	.25
	1 mo	13.6 (0.47)	12.9 (0.57)	.41
	6 mo	12.9 (0.50)	12.0 (0.62)	.29
Perceived stress score (0-40), mean (SE)^m^	BL1	16.1 (0.68)	14.1 (0.81)	.09
	6 mo	12.6 (0.82)	11.9 (1.03)	.61
Patient Health Q-2 Score (0-6), mean (SE)^n^	BL1	1.80 (0.21)	1.56 (0.24)	.47
	1 mo	1.64 (0.23)	1.39 (0.26)	.47
	6 mo	1.06 (0.21)	0.97 (0.25)	.80
EuroQol visual analogue scale (0-100), mean (SE)^o^	BL1	44.6 (2.69)	48.6 (3.07)	.36
	1 mo	64.3 (2.67)	60.5 (3.13)	.38
	6 mo	69.6 (2.57)	68.8 (3.07)	.86

Abbreviations: BL1, baseline 1 survey; BL2, baseline 2 survey; PEACE, peace, equanimity, and acceptance in the cancer experience; SE, standard error; 1 mo, 1-month follow-up; 6 mo, 6-month follow-up.

^a^ 10-item measure assessing knowledge of DT LVAD, number of correct answers.

^b^ Likert scale of 1 to 10, with 1 being “Do everything I can to live longer, even if that means having major surgery and being dependent on a machine” and 10 being “Live with whatever time I have left, without going through major surgery or being dependent on a machine.”

^c^ Patient-reported treatment decision of “I wanted the DT LVAD and decided to get it” and “I first decided not to get the DT LVAD but then decided I wanted it.”

^d^ Missing 1-month surveys: 31 (23%) in control group and 34 (30.1%) in intervention group.

^e^ Missing 6-month surveys: 37 (27.4%) in control group and 46 (40.7%) in intervention group.

^f^ Medical record report at 6 months on patients’ treatment received, LVAD or no LVAD.

^g^ 16 Items, scoring 0 to 100 with higher score indicating greater decisional conflict.

^h^ 5 Items, scoring 0 to 100 with higher score indicating greater decision regret.

^i^ 1 Item assessing preferred control in decision making, “active role” includes answers of “I prefer to make the final selection about which treatment I will receive,” “I prefer to make the final selection of my treatment after seriously considering my doctor’s opinion,” or “I prefer that my doctor and I share responsibility for deciding which treatment is best.”

^j^ 1 Item assessing actual control in decision making, “active role” includes answers of “I made the final selection about which treatment I would receive,” “I made the final selection of my treatment after seriously considering my doctor’s opinion,” or “My doctor and I shared responsibility for deciding which was treatment best for me.”

^k^ Questions 1 through 5 of 12 items, scoring 5 to 20 with higher score indicating greater acceptance of illness.

^l^ Questions 6 through 12 of 12 items, scoring 7 to 28 with higher score indicating greater struggle with illness.

^m^ 10 Items, scoring 0 to 40 with higher score indicating greater stress.

^n^ 2 Items, score of 0 to 6 with higher score indicating greater depression.

^o^ 1-Item scale, score of 0 to 100 with 0 being “worst imaginable health state” and 100 being “best imaginable health state.”

At 1 month, patient-reported treatment choice favored LVAD more in the control group than the intervention group: “wanted LVAD and decided to get it” 78.8% control, 54.4% intervention; “first decided not to get the DT LVAD but then decided he/she wanted it” 12.5% control, 5.1% intervention; “decided not to get LVAD” 1.0% control, 7.6% intervention (overall *P* < .001).

Concordance between stated values and patient-reported treatment choice (eg, values score closer to 1 combined with “wanted LVAD”) at 1-month was higher in the intervention than in the control group (Kendall’s τ correlation coefficient: control 0.17, intervention 0.48 (difference in correlation control to intervention, 0.28; 95% CI, 0.05-0.45; *P* = .01) (Figure 2). Patient-reported treatment choices were stable from 1 month to 6 months (Table 2).

**Figure 3.**
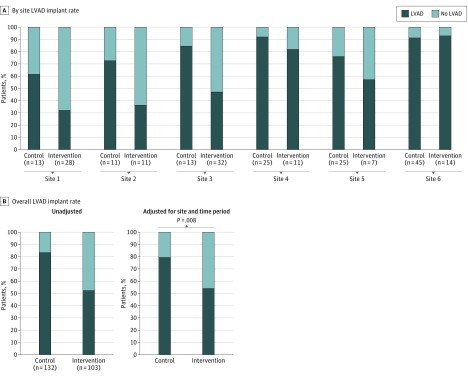
LVAD Implant by 6 Months Abbreviation: LVAD, left ventricular assist device.

By 6 months, 110 (83.3%) (adjusted rate, 79.9%) patients in the control group and 54 (52.4%) (adjusted rate, 53.9%) patients in the intervention group had undergone LVAD implantation (*P* = .008), with significant differences among sites (Figure 3). The medical team decided the patient was not eligible for a DT LVAD in 11 (8.3%) control and 25 (24.3%) intervention patients (*P* < .001). Concordance between stated values at 1 month and actual treatment received by 6 months (in contrast to the 1-month patient-reported treatment choice above) was the same in both the control and intervention groups (Kendall’s τ correlation coefficient: intervention, 0.26; control, 0.27; difference in correlation control to intervention: 0.01; 95% CI, −0.24 to 0.25; *P* = .97).

### Secondary Outcomes

At baseline and follow up, decision conflict, decision regret, control preferences, illness acceptance, stress, depression, and quality of life were not significantly different between the control and intervention groups (Table 2).

### Sensitivity Analyses

When individual sites were removed from the analysis or when the analysis was restricted only to those enrolled in the inpatient setting, the primary knowledge and values-choice concordance findings remained significantly in favor of the intervention period. In sensitivity analysis of the knowledge score that did not include the site random effect, our findings remained the same. Missing data can be viewed in Supplement 2.

## Discussion

The DECIDE-LVAD trial offers unique insights into one of the most challenging medical decisions created by modern medicine. Rather than test the efficacy of decision support tools administered by research personnel in a patient-randomized trial, this study used a pragmatic effectiveness design to assess how programmatic integration of decision aids and clinician training into standard processes of care may influence DT LVAD decision making.^22^ Through its conduct, DECIDE-LVAD created one of the largest prospective LVAD-eligible cohorts,^4^ enrolling most of the patients considered for DT LVAD during the study period, from 6 geographically diverse sites, and nearly a quarter from intensive care. In this context, the intervention was associated with better decision quality.

Decision support studies most commonly measure knowledge and decision conflict as the primary outcomes.^13^ However, central to a high-quality decision is whether the choice matches the patient’s values, goals, and preferences. Knowledge alone is insufficient to guarantee high-quality decision making, particularly for emotionally charged decisions around life and death.^41^ Similarly, nudging patients in fear and denial to address life-threatening situations—rather than providing them with false reassurances—may transiently increase feelings of conflict, anxiety, and even regret.^42^ Thus, it is not surprising to us that DECIDE-LVAD intervention did not reduce conflict at 1 month; we predicted this a priori.^43^ In contrast, we leveraged the dominant value that emerges when considering DT LVAD (ie, aggressive care to optimize survival chances vs not) and found that the intervention was associated with improved concordance between stated values and patient-reported treatment choice. This did not translate to improved concordance between stated values and actual treatment received, perhaps because DT LVAD implantation is influenced by a wide range of factors (eg, medical eligibility, presence of an adequate caregiver), many of which are not in the patient’s control.

The site-based intervention with a randomized stepped wedge roll out distinguishes the DECIDE-LVAD study from most other assessments of shared decision making. Despite strong efficacy data, uptake of decision aids in routine practice has been slow.^16^ To be successful, decision support tools must integrate easily into existing care and facilitate necessary discussions. Leveraging the formal consent and education process for LVAD, we were able to implement the DECIDE-LVAD intervention into this existing structure, while observing the programmatic transitions in all 6 sites over time. Given the importance of widespread adoption of shared decision making, pragmatic studies such as this one are needed to address real-world complexities and implementation challenges. The decrease in device implantation rates from control to intervention in 5 of the 6 sites supports the ability to influence institutional culture and decision-making processes related to major medical interventions.^44^

Unlike most prospective studies in MCS that follow patients from the time of implant, the DECIDE-LVAD study moved upstream to follow the entire population of patients formally considered for DT LVAD.^45^ By focusing on a choice rather than a specific therapy (ie, only those who have received an LVAD), DECIDE-LVAD expands insights into the patients, experiences, and processes that lead up to decisions about device implantation.^47^ We found that a significant number of patients facing high morbidity and mortality from heart failure decline DT LVAD in favor of avoiding aggressive therapies. The declination rates reported here are among patients who have agreed to undergo formal evaluation; thus, we suspect that DT LVAD declination may be more prevalent in the broader community.^48^ This is concordant with prior work showing diversity in the relative emphasis patients place on quality vs quantity of life, even when actively facing life-threatening illness.^17^ It also reinforces DT LVAD as a relatively preference-sensitive decision.

### Limitations

A number of limitations should be recognized. First, missing data were somewhat frequent and concentrated among the group of patients who did not undergo implantation of DT LVAD. Death was the most common cause of missing data, followed by withdrawal from the study, both of which are common in studies targeting patients with life threatening illness. Our missing data rates are comparable to similar study types,^43^ and our models adjusted for missing data as much as possible. Second, time trends in rapidly evolving fields are particularly problematic for the stepped wedge design.^21^ Fortunately, device technology was relatively stable between 2015 and 2017 and durable LVAD implant rates across the US plateaued somewhat during this period.^48^ Third, the phased implementation randomized the site with the lowest LVAD implant rate to spend the most time in the intervention and the site with the highest LVAD implant rate to spend the most time in the control. Linear mixed models accounting for site effects and sensitivity analyses were used to explore and diminish these differences for the patient-based effectiveness outcomes, but do not necessarily fully account for such effects.^40^ Finally, the population was mostly white males. Although enrollees were 12% more likely to be white than those excluded, the final study cohort reflects contemporary use of LVADs in the United States.^50^ This bias makes it difficult to extrapolate the findings here to decision making for women and underrepresented races/ethnicities.

## Conclusions

A shared decision-making intervention for patients considering DT LVAD—implemented programmatically, integrating novel patient decision aids, and including clinician training—was associated with a significant improvement in knowledge and an increase in concordance between stated values and patient-reported treatment choice. Although these changes did not translate to improvements in concordance between stated values and actual treatment received, patients were less likely during intervention than control to proceed to LVAD implant. These results suggest that institutional culture and processes can influence medical decisions in life-threatening illness.

## Notes

Supplement 1. **Trial Protocol**

Supplement 2. **eAppendix 1. **Pamphlet. A decision aid for Left Ventricular Assist Device (LVAD) as Destination Therapy **eAppendix 2. **Sample Surveys. Search strategy for EMBASE (using Embase.com) **eAppendix 3. **Missing Data. Flow of information through the different phases of the review **eTable. **Participant Characteristics by Missing Primary Knowledge (at Baseline 1 and Baseline 2) and Values (at 1-Month) Outcomes

## References

[ioi170138r1] Fried TR Shared Decision Making—Finding the Sweet Spot. N Engl J Med. 2016;374(2):104-106. 26760081 10.1056/NEJMp1510020

[ioi170138r2] Walsh MN, Bove AA, Cross RR, ; American College of Cardiology Foundation ACCF 2012 health policy statement on patient-centered care in cardiovascular medicine: a report of the American College of Cardiology Foundation Clinical Quality Committee. J Am Coll Cardiol. 2012;59(23):2125-2143. 22591882 10.1016/j.jacc.2012.03.016

[3] Allen LA, Stevenson LW, Grady KL, ; American Heart Association; Council on Quality of Care and Outcomes Research; Council on Cardiovascular Nursing; Council on Clinical Cardiology; Council on Cardiovascular Radiology and Intervention; Council on Cardiovascular Surgery and Anesthesia Decision making in advanced heart failure: a scientific statement from the American Heart Association. Circulation. 2012;125(15):1928-1952. 22392529 10.1161/CIR.0b013e31824f2173 PMC3893703

[ioi170138r4] McIlvennan CK, Magid KH, Ambardekar AV, Thompson JS, Matlock DD, Allen LA Clinical outcomes after continuous-flow left ventricular assist device: a systematic review. Circ Heart Fail. 2014;7(6):1003-1013. 25294625 10.1161/CIRCHEARTFAILURE.114.001391 PMC4241134

[5] Grady KL, Meyer PM, Dressler D, Change in quality of life from after left ventricular assist device implantation to after heart transplantation. J Heart Lung Transplant. 2003;22(11):1254-1267. 14585387 10.1016/s1053-2498(02)01226-3

[ioi170138r6] Elwyn G, O’Connor A, Stacey D, ; International Patient Decision Aids Standards (IPDAS) Collaboration Developing a quality criteria framework for patient decision aids: online international Delphi consensus process. BMJ. 2006;333(7565):417. 16908462 10.1136/bmj.38926.629329.AE PMC1553508

[7] Zikmund-Fisher BJ, Couper MP, Singer E, Deficits and variations in patients’ experience with making 9 common medical decisions: the DECISIONS survey. Med Decis Making. 2010;30(5)(suppl):85S-95S. 20881157 10.1177/0272989X10380466

[ioi170138r8] Feldman D, Pamboukian SV, Teuteberg JJ, ; International Society for Heart and Lung Transplantation The 2013 International Society for Heart and Lung Transplantation Guidelines for mechanical circulatory support: executive summary. J Heart Lung Transplant. 2013;32(2):157-187. 23352391 10.1016/j.healun.2012.09.013

[ioi170138r9] Iacovetto MC, Matlock DD, McIlvennan CK, Educational resources for patients considering a left ventricular assist device: a cross-sectional review of internet, print, and multimedia materials. Circ Cardiovasc Qual Outcomes. 2014;7(6):905-911. 25316772 10.1161/CIRCOUTCOMES.114.000892

[ioi170138r10] McIlvennan CK, Matlock DD, Narayan MP, Perspectives from mechanical circulatory support coordinators on the pre-implantation decision process for destination therapy left ventricular assist devices. Heart Lung. 2015;44(3):219-224. 25724116 10.1016/j.hrtlng.2015.01.012 PMC4426042

[ioi170138r11] Tulsky JA, Arnold RM, Alexander SC, Enhancing communication between oncologists and patients with a computer-based training program: a randomized trial. Ann Intern Med. 2011;155(9):593-601. 22041948 10.1059/0003-4819-155-9-201111010-00007 PMC3368370

[ioi170138r12] O’Connor AM, Wennberg JE, Legare F, Toward the ‘tipping point’: decision aids and informed patient choice. Health Aff (Millwood). 2007;26(3):716-725. 17485749 10.1377/hlthaff.26.3.716

[ioi170138r13] Stacey D, Légaré F, Lewis KB Patient decision aids to engage adults in treatment or screening decisions. JAMA. 2017;318(7):657-658. 28810006 10.1001/jama.2017.10289

[ioi170138r14] Austin CA, Mohottige D, Sudore RL, Smith AK, Hanson LC Tools to promote shared decision making in serious illness: a systematic review. JAMA Intern Med. 2015;175(7):1213-1221. 25985438 10.1001/jamainternmed.2015.1679 PMC4794743

[ioi170138r15] Gravel K, Légaré F, Graham ID Barriers and facilitators to implementing shared decision-making in clinical practice: a systematic review of health professionals’ perceptions. Implement Sci. 2006;1:16. 16899124 10.1186/1748-5908-1-16 PMC1586024

[ioi170138r16] Elwyn G, Scholl I, Tietbohl C, “Many miles to go …”: a systematic review of the implementation of patient decision support interventions into routine clinical practice. BMC Med Inform Decis Mak. 2013;13(suppl 2):S14. 24625083 10.1186/1472-6947-13-S2-S14 PMC4044318

[ioi170138r17] McIlvennan CK, Allen LA, Nowels C, Decision making for destination therapy left ventricular assist devices: “there was no choice” versus “I thought about it an awful lot”. Circ Cardiovasc Qual Outcomes. 2014;7(3):374-380. 24823949 10.1161/CIRCOUTCOMES.113.000729 PMC4081474

[18] McIlvennan CK, Jones J, Allen LA, Decision-making for destination therapy left ventricular assist devices: implications for caregivers. Circ Cardiovasc Qual Outcomes. 2015;8(2):172-178. 25759442 10.1161/CIRCOUTCOMES.114.001276 PMC4365422

[ioi170138r19] Thompson JS, Matlock DD, McIlvennan CK, Jenkins AR, Allen LA Development of a decision aid for patients with advanced heart failure considering a destination therapy left ventricular assist device. JACC Heart Fail. 2015;3(12):965-976. 26671675 10.1016/j.jchf.2015.09.007 PMC4683411

[ioi170138r20] McIlvennan CK, Thompson JS, Matlock DD, A multicenter trial of a shared decision support intervention for patients and their caregivers offered destination therapy for advanced heart failure: DECIDE-LVAD: rationale, design, and pilot data. J Cardiovasc Nurs. 2016;31(6):E8-E20. 27203272 10.1097/JCN.0000000000000343

[ioi170138r21] Mdege ND, Man MS, Taylor Nee Brown CA, Torgerson DJ Systematic review of stepped wedge cluster randomized trials shows that design is particularly used to evaluate interventions during routine implementation. J Clin Epidemiol. 2011;64(9):936-948. 21411284 10.1016/j.jclinepi.2010.12.003

[ioi170138r22] Curran GM, Bauer M, Mittman B, Pyne JM, Stetler C Effectiveness-implementation hybrid designs: combining elements of clinical effectiveness and implementation research to enhance public health impact. Med Care. 2012;50(3):217-226. 22310560 10.1097/MLR.0b013e3182408812 PMC3731143

[ioi170138r23] Pfeiffer E A short portable mental status questionnaire for the assessment of organic brain deficit in elderly patients. J Am Geriatr Soc. 1975;23(10):433-441. 1159263 10.1111/j.1532-5415.1975.tb00927.x

[24] Bass PF III, Wilson JF, Griffith CH A shortened instrument for literacy screening. J Gen Intern Med. 2003;18(12):1036-1038. 14687263 10.1111/j.1525-1497.2003.10651.x PMC1494969

[25] Fagerlin A, Zikmund-Fisher BJ, Ubel PA, Jankovic A, Derry HA, Smith DM Measuring numeracy without a math test: development of the Subjective Numeracy Scale. Med Decis Making. 2007;27(5):672-680. 17641137 10.1177/0272989X07304449

[ioi170138r26] Harris PA, Taylor R, Thielke R, Payne J, Gonzalez N, Conde JG Research electronic data capture (REDCap)--a metadata-driven methodology and workflow process for providing translational research informatics support. J Biomed Inform. 2009;42(2):377-381. 18929686 10.1016/j.jbi.2008.08.010 PMC2700030

[27] Sepucha KR, Fowler FJ Jr., Mulley AG Jr Policy support for patient-centered care: the need for measurable improvements in decision quality. Health Aff. 2004;Suppl Variation:VAR54-62. 10.1377/hlthaff.var.54 15471772

[28] University of Ottawa Ottawa Decision Support Framework. 2010 http://decisionaid.ohri.ca/docs/develop/odsf.pdf. Accessed October 20, 2017.

[ioi170138r29] Sepucha KR, Levin CA, Uzogara EE, Barry MJ, O’Connor AM, Mulley AG Developing instruments to measure the quality of decisions: early results for a set of symptom-driven decisions. Patient Educ Couns. 2008;73(3):504-510. 18718734 10.1016/j.pec.2008.07.009

[ioi170138r30] Sepucha K, Ozanne E, Silvia K, Partridge A, Mulley AG Jr An approach to measuring the quality of breast cancer decisions. Patient Educ Couns. 2007;65(2):261-269. 17023138 10.1016/j.pec.2006.08.007

[ioi170138r31] O’Connor AM Sample Tool: Values (Tamozifen). 1999; https://decisionaid.ohri.ca/eval_values.html. Accessed August 4, 2017.

[ioi170138r32] O’Connor AM Validation of a decisional conflict scale. Med Decis Making. 1995;15(1):25-30. 7898294 10.1177/0272989X9501500105

[ioi170138r33] Brehaut JC, O’Connor AM, Wood TJ, Validation of a decision regret scale. Med Decis Making. 2003;23(4):281-292. 12926578 10.1177/0272989X03256005

[ioi170138r34] Janz NK, Wren PA, Copeland LA, Lowery JC, Goldfarb SL, Wilkins EG Patient-physician concordance: preferences, perceptions, and factors influencing the breast cancer surgical decision. J Clin Oncol. 2004;22(15):3091-3098. 15284259 10.1200/JCO.2004.09.069

[ioi170138r35] Mack JW, Nilsson M, Balboni T, Peace, Equanimity, and Acceptance in the Cancer Experience (PEACE): validation of a scale to assess acceptance and struggle with terminal illness. Cancer. 2008;112(11):2509-2517. 18429006 10.1002/cncr.23476 PMC3809101

[ioi170138r36] Cohen S, Kamarck T, Mermelstein R A global measure of perceived stress. J Health Soc Behav. 1983;24(4):385-396. 6668417

[ioi170138r37] Kroenke K, Spitzer RL, Williams JB The Patient Health Questionnaire-2: validity of a two-item depression screener. Med Care. 2003;41(11):1284-1292. 14583691 10.1097/01.MLR.0000093487.78664.3C

[ioi170138r38] Brooks R EuroQol: the current state of play. Health Policy. 1996;37(1):53-72. 10158943 10.1016/0168-8510(96)00822-6

[ioi170138r39] Barry MJ, Fowler FJ Jr, Mulley AG Jr, Henderson JV Jr, Wennberg JE Patient reactions to a program designed to facilitate patient participation in treatment decisions for benign prostatic hyperplasia. Med Care. 1995;33(8):771-782. 7543639 10.1097/00005650-199508000-00003

[ioi170138r40] Hussey MA, Hughes JP Design and analysis of stepped wedge cluster randomized trials. Contemp Clin Trials. 2007;28(2):182-191. 16829207 10.1016/j.cct.2006.05.007

[ioi170138r41] Sepucha KR, Borkhoff CM, Lally J, Establishing the effectiveness of patient decision aids: key constructs and measurement instruments. BMC Med Inform Decis Mak. 2013;13(suppl 2):S12. 24625035 10.1186/1472-6947-13-S2-S12 PMC4044563

[ioi170138r42] Politi MC, Clark MA, Ombao H, Dizon D, Elwyn G Communicating uncertainty can lead to less decision satisfaction: a necessary cost of involving patients in shared decision making? Health Expect. 2011;14(1):84-91. 20860780 10.1111/j.1369-7625.2010.00626.x PMC3010418

[ioi170138r43] Korteland NM, Ahmed Y, Koolbergen DR, Does the use of a decision aid improve decision making in prosthetic heart valve selection? A multicenter randomized trial. Circ Cardiovasc Qual Outcomes. 2017;10(2):e003178. 28228452 10.1161/CIRCOUTCOMES.116.003178

[ioi170138r44] Nelson EC, Godfrey MM, Batalden PB, Clinical microsystems, part 1. The building blocks of health systems. Jt Comm J Qual Patient Saf. 2008;34(7):367-378. 18677868 10.1016/s1553-7250(08)34047-1

[ioi170138r45] Estep JD, Starling RC, Horstmanshof DA, ; ROADMAP Study Investigators Risk assessment and comparative effectiveness of left ventricular assist device and medical management in ambulatory heart failure patients: results from the ROADMAP study. J Am Coll Cardiol. 2015;66(16):1747-1761. 26483097 10.1016/j.jacc.2015.07.075

[46] Ambardekar AV, Thibodeau JT, DeVore AD, Discordant perceptions of prognosis and treatment options between physicians and patients with advanced heart failure. JACC Heart Fail. 2017;5(9):663-671. 28822745 10.1016/j.jchf.2017.04.009 PMC5609812

[ioi170138r47] Ambardekar AV, Forde-McLean RC, Kittleson MM, High early event rates in patients with questionable eligibility for advanced heart failure therapies: Results from the Medical Arm of Mechanically Assisted Circulatory Support (Medamacs) Registry. J Heart Lung Transplant. 2016;35(6):722-730. 26987599 10.1016/j.healun.2016.01.014 PMC4917444

[ioi170138r48] Kirklin JK, Naftel DC, Pagani FD, Seventh INTERMACS annual report: 15,000 patients and counting. J Heart Lung Transplant. 2015;34(12):1495-1504. 26520247 10.1016/j.healun.2015.10.003

[49] Lewis EF, Johnson PA, Johnson W, Collins C, Griffin L, Stevenson LW Preferences for quality of life or survival expressed by patients with heart failure. J Heart Lung Transplant. 2001;20(9):1016-1024. 11557198 10.1016/s1053-2498(01)00298-4

[ioi170138r50] McIlvennan CK, Lindenfeld J, Kao DP Sex differences and in-hospital outcomes in patients undergoing mechanical circulatory support implantation. J Heart Lung Transplant. 2017;36(1):82-90. 27773454 10.1016/j.healun.2016.08.013 PMC7061938

[51] Meeteren JV, Maltais S, Dunlay SM, A multi-institutional outcome analysis of patients undergoing left ventricular assist device implantation stratified by sex and race. J Heart Lung Transplant. 2017;36(1):64-70. 27793517 10.1016/j.healun.2016.08.027

