# Supplementary material for: Effectiveness of an Intervention Supporting Shared Decision Making for Destination Therapy Left Ventricular Assist Device: The DECIDE-LVAD Randomized Clinical Trial
Source: JAMA Intern Med. 2018 Feb 26;178(4):520–9. doi: 10.1001/jamainternmed.2017.8713 (PMC5876922; doi:10.1001/jamainternmed.2017.8713)
Supplement: Supplement 1. — Trial Protocol [file jamainternmed-178-520-s001.pdf]

COMIRB Protocol

COLORADO MULTIPLE INSTITUTIONAL REVIEW BOARD
CAMPUS BOX F-490 TELEPHONE: 303-724-1055 Fax: 303-724-0990

Protocol #: 14-2102
Project Title: A multicenter trial of a shared decision support intervention for patients and their caregivers offered destination therapy for end-stage heart failure: DECIDE-LVAD
Principal Investigator: Larry Allen, MD, MHS
Version Date: 04/11/2016

I. Hypotheses and Specific Aims:
The research team proposes to understand the effectiveness and implementation of a shared decision support process for advanced heart failure patients considering left ventricular assist device for destination therapy (DT LVAD). A standard decision support process has been developed, which includes patient/caregiver-directed decision aids and a clinician-directed decision training that will be used to help patients make the most educated and values-driven decision possible. This new decision support process will be adopted at 6 medical centers with an LVAD program. The implementation of this new process will occur in shifts. This study aims to understand if the new decision support process helps patients and caregivers make decisions that are more knowledgeable and values-concordant, compared to the current education process. The current education process around DT LVAD consists of communication with doctors, LVAD coordinators and other clinical staff. No standard education process exists across all LVAD programs, and the only available education materials (videos, pamphlets, etc.) are industry created, and thus biased toward accepting DT LVAD (see "Preliminary Studies" below).

Thus, we will collect survey data from patients and caregivers while the current process is used, and then from patients and caregivers when the new process is in place. This will help us understand the reach and effectiveness of the new process on patient and caregiver experiences with DT LVAD decision-making. Additionally, we will interview some of the enrolled caregivers to obtain further details about the process.

All 6 medical centers have agreed to implement this new decision support process as standard care during the study intervention period—thus, all patients being considered for DT LVAD will receive the decision support intervention regardless of their enrollment in this study.

Aim: Evaluate the reach and effectiveness of the DT LVAD shared decision support process to improve *patient* and *caregiver* experiences.

II. Background and Significance:
Technologies for Advanced Chronic Illness are Growing
Patients with chronic progressive illness face an increasing array of decisions about invasive and potentially life-prolonging technologies.[1] Meanwhile, the number of people in the U.S. over the age of 65 is increasing from 35 million in the year 2000 to an expected 87 million in 2050. This demographic, termed the "silver tsunami" by the Institute of Medicine,[2] will likely have multiple chronic medical conditions and are more likely to develop end-stage illness. These trends in chronic progressive multi-morbidity and the availability of invasive treatment options are already placing a significant burden on the health care system, the communities, and the families who help care for these vulnerable patients.[1]

For patients and their loved ones, decisions about whether or not to pursue invasive technologies in the setting of chronic progressive illness are arguably the most complicated applications of the well-intended PCORI question: "What are my options and what are the benefits and harms of those options?" Well-meaning clinicians are often ill-prepared for the complicated and

lengthy conversations that these decisions require. Our preliminary data (see Section B.2) demonstrate that the complexity and related emotion around chronic progressive illness create a scenario where patients *and caregivers* are often too afraid to discuss alternatives or hear about complications and burdens. Learning how to help patients *and caregivers* make high quality decisions is an area in desperate need of attention.

Therefore, we aim to address this desperate need by studying a novel shared decision support intervention for patients with one of the most common chronic progressive illnesses—heart failure—who are offered a prototype invasive technology—i.e. destination therapy left ventricular assist device (DT LVAD).

DT LVAD is Growing

LVADs are effectively a partial artificial heart. In heart failure, the heart is too weak to pump blood to meet the needs of the body. Very simply, LVADs pull blood from the left ventricle and pump into the aorta at a higher pressure in order to increase blood flow. LVADs must be connected to batteries or an electrical outlet outside of the body through a hole in the skin of the belly.

LVADs are becoming a mainstream treatment option for people dying from heart failure.[3] Previously, LVADs were developed as a “bridge” to stabilize people waiting for a heart transplant. However, a decade ago, the Food and Drug Administration approved LVADs as “destination therapy” for people with end-stage heart failure who are too old or too sick from other medical problems to get a heart transplant. As device technology improves, the number of older patients with chronic heart failure continues to increase and DT LVAD use promises to grow. An estimated 150,000-250,000 patients each year could be eligible for DT LVAD[4] from the more than 6,000,000 in the U.S. with chronic heart failure.[5,6]

Benefits, Risks and Burdens of DT LVAD

Due to technological improvements in recent years, clinical outcomes are improving.[7] For patients with end-stage heart failure, 2-year survival is dismal at <10%,[8-10] whereas with DT LVAD, 2-year actuarial survival is now >60%, with some patients living for many years.[7] For those patients who do survive with DT LVAD to 2 years, formal measures of quality of life nearly double from pre-implantation.[11]

These striking benefits come with a host of serious risks. Even with the newest technology, more than half of patients experience a major adverse event within the 2 years after implantation, including reoperation to replace a malfunctioning pump (~10%), disabling stroke (~11%), life-threatening infection (~33%), and major bleeding (~25%). Patients are rehospitalized an average of 1-2 times per year for these adverse events and other complications (See section B.2 Project #6 for a discussion of our systematic review of patient outcomes following LVAD implantation).

Irrespective of potential complications, LVADs require a high degree of ongoing device care.[12] Interruption of electrical input to the pump typically results in rapid loss of consciousness and death. Additionally, LVAD therapy includes daily self-care (e.g., controller self-tests, charging batteries, and driveline exit site dressing changes), safety precautions (e.g. no emersion in water, using a shower kit, traveling precautions), the ability to manage alarms, and cost of device care (which is being increasingly shifted from third-party payers to patients). Our shared decision support intervention uses evidence-based strategies of risk communication to present both the risks and benefits in a way that patients and caregivers can understand.

DT LVAD Eligible Patients Have Multiple Chronic Conditions

The most common reasons for heart transplant ineligibility, and consequently DT status, are advanced age and non-cardiac medical illness.[13] Therefore DT LVAD candidates are generally older and sicker than patients being considered for bridge-to-transplant LVAD and heart transplantation. Even after successful DT LVAD implantation without serious complications, most patients will have other non-cardiac comorbidity or ongoing frailty, particularly if these health factors are not carefully considered prior to surgery.[14] Although DT LVAD is often described as “life saving”, semantically the phrase “potentially life-prolonging” better captures the ongoing medical issues and a >50% death rate over the next 5 years. Our shared decision support intervention carefully frames DT LVAD within this context.

High-Level of Caregiver Involvement in DT LVAD
Although caregivers form a critical component of the care of patients with chronic disease, their role is particularly central and formalized in the setting of DT LVADs. Most LVAD programs have a mandatory requirement for a dedicated and capable caregiver in order for a patient to be eligible for a DT LVAD. This level of caregiver responsibility comes with a host of potential burdens.[15,16] A study of 27 spouses of patients with LVADs reported that 26% met clinical criteria for post-traumatic stress disorder.[17] Caregivers in 4 separate studies described the experience similar to being a "new mom again caring for a newborn".[16] Another study raised concerns that caregivers are not well prepared for the terminal nature of DT LVADs.[18]

As the PCORI announcement states, "Research on doctor-patient communication has focused primarily on the doctor-patient dyad, but still little is known about the potential role of the patient's family members or significant others in shaping the decision-making process." <u>Our shared decision support intervention takes special care to target both patients and caregivers alike, and we aim to improve outcomes for both groups</u>.

Critical Need for Novel Decision Support for Patents Offered DT LVAD
Given these complex tradeoffs, necessary components for shared decision making around DT LVAD must include optimal patient selection, extensive informed consent, adequate time to review expected risks, benefits, and burdens, and a strong grounding in patients' and caregivers' goals and values. Our group worked diligently to outline and promote such concepts in a 2012 American Heart Association Scientific Statement entitled "Decision Making in Advanced Heart Failure".[19]

Our preliminary work (see below) shows concerning gaps in the current DT LVAD decision-making process: the current decision process is highly variable across institutions; existing consents and industry materials are too complicated and optimistically biased; while thankful, caregivers carry significant burdens; and the majority of patients do not perceive there to be a decision, with DT LVAD framed as their only reasonable option. There is an urgent need to improve the shared decision making process for all high-stakes, medically complex decisions in general and DT LVAD in particular. It is hard to overstate the significance of learning how to help patients with chronic progressive illness and their caregivers make decisions about highly invasive and potentially life-prolonging interventions. If not used properly, these "options" have the potential to harm patients, burden families, and bankrupt our nation.[1]

One way forward is to make sure that these decisions are of a high quality (informed, concordant with patient and caregiver values and goals).[20-22] Unfortunately, for many major medical decisions involving technologies, it does not appear that appropriate education, consent, and shared decision making is happening.[23,24] Perhaps even more concerning are reports that patients often receive care that they would not want.[25] Older adults with chronic conditions—like those being considered for DT LVAD—provide additional challenges in that they are more likely to be cognitively impaired, have lower health literacy, or be passive in their decision making.[26-28]

III. Preliminary Studies/Progress Report:

We have conducted a series of studies in the area of DT LVAD decision making that clarify the major barriers to high quality decision making in this situtation (see **Table 1** below), and provide the preliminary needs assessment (see Ottawa Decision Support Framework section B.4.b) that guided the development of the DT LVAD decision support intervention. The importance of this proposal is highlighted by our extensive work with patient, caregiver, and provider stakeholders.

<u>Project #1: Poor Quality of Existing DT LVAD Decision Support Materials – A Review</u>
In July 2013, we performed a cross-sectional review of internet, print, and multimedia resources available to patients considering LVADs.[29] We identified and reviewed 77 tools for content, accuracy, readability. For those few tools that explicitly discussed decisions, we further reviewed them for adherence to International Patient Decision Aid Standards (IPDAS) criteria and we assembled a focus group to assess for bias. We concluded that while many resources exist, the content is overly optimistic, often incorrect or outdated, and biased, as most materials are

distributed by manufacturers interested in selling their device or hospital programs looking to grow procedural volumes.

Project #2: Programmatic Variation and Deficiencies – LVAD Coordinator Qualitative Interviews (N=18)
LVAD coordinators, typically nurses or nurse practitioners, are central to the broad medical team that interacts with patients considering DT LVAD. These professionals are responsible for the majority of education pre-LVAD. Therefore, they are well positioned to comment upon the decision making process. In late 2012, we interviewed 18 LVAD coordinators from across the U.S. to characterize the range of decision support currently offered to patients considering DT LVAD. Coordinators identified 3 tools commonly used by their programs. Coordinators said this was a major and personal decision requiring an artful approach: *"It's sometimes hard to walk that line to not scare them but not paint a rainbow and butterflies picture."* They also noted that many patients do not view it as a decision at all. Overall, they endorsed a more standardized and balanced decision process and they expressed a desire for more understandable materials.[30]

Table 1: Summary of Preliminary Data Projects Surrounding DT LVAD Decision Making

Project	Major Finding
1: A review of existing patient materials	Existing tools are overly optimistic, biased, and often contain incorrect or outdated data.
2: Qualitative interviews: LVAD coordinators	LVAD coordinators endorsed a DT LVAD decision process that is more standardized, iterative, and balanced, and they expressed a desire for better quality materials.
3: Qualitative interviews: Patients offered DT LVAD	There is a strong dichotomy approach, with some patients deciding automatically based on a desire to live and others reflecting on quality of life implications.
4: Qualitative meta-synthesis of LVAD caregivers	Caregivers go through an early phase of fear and anxiety followed by a more complicated phase characterized by hypervigilence and varied coping strategies.
5: Qualitative interviews: Caregivers of patients offered DT LVAD	In addition to the fear, anxiety, hypervigilence and varied copied strategies identified in #4, caregivers also feel a tension between burdens and gratitude.
6. Systematic review of LVAD outcomes	Consistent improvements in function and quality of life were marked, although survival was 47% at 4 years and adverse events were experienced by the majority of patients.

Project #3: Patient Interviews with DT LVAD Acceptors (n=15) and Decliners (n=7)
We interviewed 22 patients who had gone through DT LVAD decision making.[31] We found a strong dichotomy in the decision processes that patients used to make this decision. Some patients described an "automatic" decision making process characterized by a fear of dying and an overriding desire to live as long as possible. All patients in this group accepted the DT LVAD therapy. In contrast, the second group (including all 7 decliners) described a more "reflective" process in their approach to decision making. They weighed risks, benefits, and burdens, during which both quality and quantity of life were considered. For most patients, this was a highly emotional decision. To all, a cognitive weighing of risks and benefits took a secondary role to the dominant value trade-off between self-preservation versus quality of life considerations.

Project #4: A Qualitative Meta-Synthesis of Caregivers' Experiences
We performed a qualitative meta-synthesis of 8 existing qualitative studies identified during a systematic search.[16] This study illuminated the challenges that caregivers experience, including fear and anxiety in the early phase followed by a more complicated phase of hypervigilence, varied coping strategies, and often gratitude that their loved one was still alive. Caregivers of patients with DT LVADs had significant uncertainty and denial about the future: *"hopefully they can just keep replacing the device"*.[16]

Project #5: Caregiver Interviews, Current (N=10) and Bereaved (N=7)
We also interviewed 17 caregivers of patients who had undergone DT LVAD. In addition to echoing the importance of emotion and fear of death in this decision process, caregivers highlighted the complicated tensions they feel between wanting to respect the patient and wanting the patient to live. Additionally, they expressed a tension between burden and gratitude as time passed. Notably, the caregivers felt that decision support materials specifically designed to their position would be helpful (*data not yet published*).

Project #6: A Systematic Review of Patient-Oriented LVAD Outcomes
In order to establish an accurate foundation of knowledge to inform the decision aid and limit criticism from manufacturers and other potentially conflicted entities, we conducted a formal systematic review of clinical outcomes for DT LVAD. We identified 55 articles discussing patient-oriented outcomes. Consistent improvements in functional class and quality of life were marked, although even with DT LVAD, survival was 47% at 4 years and adverse events were experienced by the majority of patients.[32]

IV. Research Methods

A. Outcome Measure(s):

Reach: Reach is defined as the proportion of the target population who receive the intervention. We will assess the percentage of patients and caregivers that received the video decision aids. The study coordinator will complete a checklist detailing what materials the patient and caregiver viewed during the education process and how they viewed them (i.e. DVD on hospital TV or at home online; completed exercises on back of pamphlet, etc.).

Effectiveness:

- Primary Outcome: Decision quality is an essential element of the Ottawa Decision Support Framework, defined as "the extent to which the implemented decision reflects the considered preferences of a well-informed patient."[21,33] By this definition, a decision is "a quality decision" if the treatment chosen is concordant with a knowledgeable patient's values.[21] Measures of decision quality have been proposed as components of the pay for performance agenda,[21] and the Patient Protection and Affordable Care Act calls for "the development of quality measures that allow for the assessment of the experience, quality, and use of information provided to and used by patients..."[34] Decision quality measures consist of 2 domains: knowledge and values.
    - Knowledge: Consistent with methods developed by Sepucha et al.,[35] we have developed a knowledge measure. To assure content validity, we have surveyed several patients, caregivers, LVAD coordinators and physicians to determine the key knowledge items. From this list of key knowledge items, we have developed a 10-item knowledge measure. We will use this measure to test both patient and caregiver knowledge twice at baseline and once at 1-month and 6 months follow-up.
    - Values: We will calculate concordance between patients' and caregivers' values and the treatment they choose according to the validated methods of Sepucha et al.[35] Specifically, we will measure the values-clarity sub-scale of the decision conflict measure (test-retest reliability and Cronbach's alpha > 0.78, correlated with knowledge, regret, and treatment discontinuance). We will also explore the prevailing value dichotomy clearly evident in our patient and caregiver qualitative research: "living longer even if it means getting an invasive therapy" versus "not living as long and avoiding an invasive therapy." Dr. Matlock has developed and successfully used a similar values-concordance question in his work on implantable defibrillators. We will use this measure to test both patient and caregiver knowledge at baseline and 1-month and 6 months follow-up.

- Secondary outcomes: Additionally, decision conflict, decision regret, control preferences, illness acceptance, caregiving preparedness, caregiver involvement in decision-making, quality of life, stress, depression/anxiety, literacy, numeracy, and cognitive function will be collected from patients and caregivers at baseline, then 1 month and 6 months after enrollment. These will help us understand satisfaction with the decision and well-being at each time point. Interviews with caregivers will allow us to further supplement the quantitative data with details and descriptions of the process and caregiving in order to have a better understanding of stress, preparedness, burden, and satisfaction.

B. Description of Population to be Enrolled:

Participants of this study will include:

1. Adult patients who have advanced heart failure and are being evaluated for DT LVAD.
2. Caregivers of patients who are being evaluated for DT LVAD.

Due to the invasive and complex nature of DT LVAD, a mandatory evaluation process already exists that formally incorporates patient and caregiver education. Within this existing system of evaluation, all patients and their caregivers will be approached and offered enrollment in this study.

3. Clinicians involved in the enrolled patient's medical care.

Exclusion Criteria:

- Under 18 years of age
- Non-English speaking (decision aids and study assessments are in English only)
- Unable to consent
- Prisoners

C. Study Design and Research Methods

We propose to test the effectiveness and implementation of a shared decision support process for DT LVAD. Six DT LVAD programs from across the U.S. will participate in a stepped-wedge randomized study design.[36] In this design, each site participates in both the control and intervention phase, with the timing of the transition randomly assigned. Sites all begin in the control phase, where usual care consists of the current education, decision making, and informed consent process. When sites reach their randomly assigned time to transition to the intervention, their coordinators and key staff will participate in communication training and decision coaching. The pamphlet and video decision aid will be formally integrated into the existing education, decision making, and informed consent process. In both pre- and post-phases, we will enroll patients and caregivers and survey them prior to their DT LVAD decision and twice after their DT LVAD decision (baseline, 1-month, and 6-months). The surveys will determine the decision support process' effect on decision quality and a host of secondary outcomes. The clinician directly involved in the medical care of each enrolled patient will also be recruited to complete a brief survey about the patient at baseline.

Due to the invasive and complex nature of DT LVAD, a mandatory evaluation process already exists that formally incorporates patient and caregiver education. Additionally, Medicare's recent Decision Memo49 reads that "Beneficiaries receiving VADs for DT must be managed by an explicitly identified cohesive, multidisciplinary team … to offer optimal patient-centered care. Collectively the team must ensure that patients and caregivers have the knowledge and support necessary to participate in shared decision making." Within this existing system of evaluation, all patients and their caregivers will be approached and offered enrollment in this trial.

It's important to note that all patients and caregivers will receive the new decision support process, regardless of their decision to participate in the study. All 6 sites in this project have agreed to implement the decision aid materials for all patients at the start of intervention—thus, the new decision support process will become the standard of care and education. Thus, the participants are consenting to the data collection only – participants are agreeing to complete the surveys and allow for medical record review.

Recruitment: To identify potential participants, we will employ a three-step process.

- Step 1: A study team member at each site (PI, study coordinator), who will have a clinical relationship with all patients due to the nature of his/her clinical position, will screen patients who are referred for DT LVAD. Once potentially eligible patients are identified, a study team member will review the chart to assure that the referred patient meets eligibility criteria and is being considered for DT LVAD.
- Step 2: If patients are potentially eligible after step 1, a study team member will contact the clinical staff to obtain verification that this is indeed a DT LVAD-eligible patient who could potentially be enrolled into the study and the study team member will obtain permission to approach the patient.
- Step 3: At that time, a study team member will call or approach the patient and introduce the study and invite the patient and caregiver to participate. Interested patients and caregivers will be asked what would be the best time for them to meet. Patients and caregivers agreeing to participate will sign the consent form. If either a patient or a caregiver refuses to participate, we will still offer participation to their counter-part (i.e. patient refuses, we would still enroll the caregiver if they were interested).

Clinician participants will be recruited in-person, through email, or over the phone by a study team member. The clinician directly involved in the enrolled patient's care will be recruited.

Data Collection: All enrolled patients will have medical record review at baseline, 1-month, 6-months, and 12-months after enrollment. Study personnel will review the medical record of the patient to determine health status, which will include looking at patient's reported comorbidities, lab results, procedures, functional status, medication list, medical evaluation for DT LVAD, treatment choice, and health status over time. Patients and caregivers will be asked to complete 3 sets of surveys: (1) baseline survey, (2) 1-month follow-up, and (3) 6-month follow-up. Participants will be provided with a $25 gift card after each set of surveys.

- Baseline Survey: Baseline surveys should be completed prior to DT LVAD education, ideally within 48 hours of enrollment. At the initial visit, participants will complete the demographics section, a mental status questionnaire (SPMSQ), literacy screen (REALM-R), and numeracy test, as well as: DT LVAD knowledge questionnaire, DT LVAD decision quality values, Decision Conflict, Perceived Stress Scale, Patient Health Questionnaire-2,, the PEACE Illness Acceptance Measure, quality of life (EQ Visual Analogue Scale), Control Preferences Scale, and the Preparedness for Caregiving survey. Study coordinators will report if and how patients and caregivers viewed education materials, with a checklist of items. Additionally, participants will fill out the Decision Conflict and Knowledge questionnaires a second time, and the Acceptability Questionnaire about educational materials, immediately after viewing the DT LVAD educational materials, so we can get a rapid response to the education and assess its effectiveness more accurately.
- 1-month Follow-up: About one month after participants make a decision on receiving or declining a DT LVAD, they will complete another set of surveys to measure DT LVAD knowledge, decision quality- values, decision conflict, decision regret, decision participation and preference, depression, illness acceptance, quality of life, caregiver preparedness, and caregiver involvement in decision-making. We will also record the patient's decision on DT LVAD and their medical status (i.e. currently hospitalized or discharged, death). For bereaved caregivers, we will also measure their satisfaction with the end of life experiences and care of the patient.
- 6-month Follow-up: Six months after participant enrollment, they will complete another set of surveys to measure DT LVAD knowledge, decision quality- values, decision conflict, decision regret, decision participation and preference, perceived stress, depression, illness acceptance, quality of life, caregiver preparedness, and caregiver involvement in decision-making. We will also record the patient's medical status (i.e. currently hospitalized or

discharged, death). For bereaved caregivers, we will also measure their satisfaction with the end of life experiences and care of the patient.

	PATIENT			CAREGIVER		
	Baseline	1-Month Follow-Up	6-Month Follow-Up	Baseline	1-Month Follow-Up	6-Month Follow-Up
**Demographics**	X			X		
**Cognitive Function**: Short Portable Mental Status Questionnaire	X			X		
**Literacy**: Realm-R	X			X		
**Numeracy**: Subjective Numeracy Scale	X			X		
**Quality of Life**: EQ Visual Analogue Scale	X	X	X			
**DT-LVAD Decision Quality Knowledge**	X	X	X	X	X	X
**DT-LVAD Decision Quality Values**	X	X	X	X	X	X
**Decision Conflict**	X	X	X	X	X	X
**Decision Regret**		X	X		X	X
**Control Preferences**: Control Preferences Scale – Preferred	X	X				
**Control Preferences**: Control Preferences Scale – Actual		X				
**Illness Acceptance**: PEACE Illness Acceptance Scale	X	X	X			
**Depression**: Patient Health Questionnaire-2	X	X	X	X	X	X
**Stress**: Perceived Stress Scale	X		X	X		X
**Caregiver Preparedness**: The Preparedness for Caregiving Scale				X	X	X
**Caregiver Involvement in Decision Process**: Family Satisfaction with Care Questionnaire					X	
**Bereaved Caregiver's Satisfaction with End of Life Care**: CANHELP – Bereavement [for bereaved caregivers only]					[X]	[X]
**Acceptability of Educational Materials**: Acceptability Questionnaire	X			X		

All surveys will preferably be completed in-person, either while the patient is hospitalized or before/after an outpatient clinic visit. In-person surveys can be completed either by the participant themselves or orally with the study coordinator, in order to make the completion of the survey as easy as possible for the participant. If surveys are unable to be completed in-person, participants can complete them either orally over the telephone with the study coordinator or through mail or email.

Participants can choose to be involved in portions of the study and refuse other parts without penalty. For instance, a patient can choose to allow medical record review, but decide to not complete any surveys.

Data will be collected through paper surveys and then entered electronically on REDCap, the University of Colorado IRB-preferred system. Data quality checks will be performed every few months during periods of active data collection.

Interview: Some of the enrolled caregivers will be asked to complete an interview using a semi-structured interview guide to provide more information about the decision-making and caregiving process, stress, preparedness and satisfaction. These interviews will be conducted either in-person or over the telephone by study team members, audio recorded, and then transcribed for analysis. Audio recordings, interview notes, and transcriptions will be stored on the University of Colorado secure server, accessible only by study team members. Caregivers can refuse participation in the interview while still maintaining their involvement in the other parts of the study.

Clinician Survey*:* Clinicians will complete the "Provider Survey" at baseline only. The clinician will answer two questions about their opinions on the enrolled patient's treatment options. This will help us understand the clinicians' view on the treatment decision in context of the patient and caregivers' opinions and decision. This survey will either be collected on paper or through a REDCap survey. The Study Coordinator can either approach the clinician in-person to complete the paper survey or email the clinician a link to the REDCap survey to be completed online. In both cases, the survey will be completed anonymously, where no clinician identifiers will be recorded on the survey itself or at any time. The survey responses will be connected to the enrolled patient participant's unique study ID number only.

D. Description, Risks and Justification of Procedures and Data Collection Tools:

Participants: We believe that this study is minimal risk to all subjects involved. Participants are only answering surveys and interview questions and allowing for medical record review, thus there is no physical risk to them. While there is the possibility that the participants may be upset by some of the survey/interview questions because it discusses the possible futures that patients with heart failure have, studies of patients with serious illness show that patients actually are not upset by such studies and can find them helpful. Further, patients may terminate participation at any point.

There are few risks to the patient or caregiver for the proposed study, and these risks relate primarily to the psychological nature of discussing the patient's serious illness. The research assistant will stop any data collection where the caregiver or patient appears to become emotionally upset, and all patients/caregivers will also be told they can terminate study participation at any point. A protocol for addressing extreme emotional reactions will be in place should such a situation arise.

Data Safety: Data collection and storage has been planned where it is improbable that participant confidentiality will be breached. All patients will be given a unique identification number, and study data and identifiable information will always be kept separate. REDCap, the COMIRB-preferred system, will be used to store all data, and access will be limited to study personnel only. All paper documents will be stored in a secure and locked file cabinet in a secure and locked office building – again, all study data paperwork will be stored separate from paperwork with identifiable information (i.e. signed consent forms).

E. Potential Scientific Problems:

The biggest potential scientific problems are related to lack of enrollment and lack of retention. However, we are currently working on a local pilot study (COMIRB #14-1127) meant to discover potential problems with the implementation and work those out prior to this multi-center trial. Having practice with recruiting and retaining patients through the pilot, we are confident that we are utilizing the most successful approach with this patient population. We will teach our lessons

learned to the other project sites during a kickoff meeting, which will occur prior to participant enrollment.

F. Data Analysis Plan:

We will assess the process' effect on decision quality for the patients and caregivers. We will use the following measures:

- Acceptability: We will measure acceptability of education materials by using a modified version of decision aid acceptability developed by Barry et al.[37]
- Feasibility: To evaluate feasibility, we will explore participation rates and adherence to the study protocol. Additionally, we will collect information on how the patient and caregiver viewed the education materials, through the study coordinator reported checklist.
- Knowledge: Consistent with methods developed by Sepucha et al.,[38] we have developed a knowledge measure. To assure content validity, we have surveyed several patients, caregivers, LVAD coordinators and physicians to determine the key knowledge items. From this list of key knowledge items, we have developed a 10-item knowledge measure.
- Decision Quality Value: To understand if participants' values are concordant with the treatment decision, this measure was developed using Sepucha et al. and previous work on ICD decision making by Co-I Dr. Dan Matlock.
- Decision Conflict: We will use the validated 15-item decision conflict measure developed by O'Connor et al.[39]
- Decision Regret: We will use the validated 5-item decision regret scale.[40]
- Control Preferences Scale (preferred and actual): We will measure the participants' preferred role in decision making using the validated Control Preferences Scale and their actual control participation. [41]
- Perceived Stress Scale: To assess the stress patients and caregivers feel during the decision making process.[42]
- *Patient Health Questionnaire-2*: To assess depression in patients and caregivers at baseline and after their decision.[43]
- PEACE Illness Acceptance Measure: For patients, we will assess patients' acceptance of their current heart failure illness and whether that impacts their decision to accept or decline DT LVAD.[44]
- Quality of Life (EQ Visual Analogue Scale): For patients, we will assess their self-reported quality of life score through only the Visual Analogue Scale portion of the EQ5D, both pre and post decision.[45]
- The Preparedness for Caregiving Scale: For caregivers, we will assess their level of preparedness, both mentally and physically, pre and post decision.[46]
- .[47]
- Family Satisfaction with Care: For caregivers, we will assess their satisfaction with the decision making process and their involvement.[48]

Quantitative questionnaire data will be scored and summarized according to the methods previously validated and published for each questionnaire. Significance for differences between quantitative statistics will be determined using Chi-square tests.

Analyses of effectiveness will use a repeated measures mixed model. This strategy allows for partially incomplete data (e.g. missing follow-up or one of the patient/caregiver pair) and relaxes missing data assumptions to missing at random conditional on observed data. Prior to these analyses, we will contrast the participants in the 2 phases of the study, identifying any patient/site characteristics that are unbalanced. If more than 3-5 variables are identified, we will develop a propensity score for the likelihood of being in the intervention phase. Each analysis model will include an indicator variable for the intervention phase, indicators for each of the sites and the variables identified above.

Basic demographics of the participants will be summarized quantitatively in a way that characterizes the study participants but cannot be traced to individuals (e.g. age ranges by decades, no specific dates).

Recorded interviews with caregivers will be evaluated using standard qualitative methods, including thematic development and member checking.

G. Summarize Knowledge to be Gained:

Formal study of the implementation will promote widespread dissemination of this DT LVAD shared decision support. This study will potentially help us improve the process for patients and caregivers going through DT LVAD consideration. This would benefit all future patients and families facing this decision, and would help contribute superior education materials to the current decision-making environment. In the broad view, this study will inform the science of decision support implementation for many invasive therapies in the setting of other chronic progressive illness.

H. References:

1. Goldman DP, Shang B, Bhattacharya J, et al. Consequences of health trends and medical innovation for the future elderly. *Health Aff. (Millwood).* 2005;24 Suppl 2:W5R5-17.
2. Institute of Medicine. Retooling for an Aging America: Building the Health Care Workforce. Washington, DC: The National Academies Press; 2008: http://www.iom.edu/CMS/3809/40113/53452.aspx
3. Kirklin JK, Naftel DC, Kormos RL, et al. Fifth INTERMACS annual report: risk factor analysis from more than 6,000 mechanical circulatory support patients. *J. Heart Lung Transplant.* Feb 2013;32(2):141-156.
4. Miller LW, Guglin M. Patient selection for ventricular assist devices: a moving target. *J. Am. Coll. Cardiol.* Mar 26 2013;61(12):1209-1221.
5. Go AS, Mozaffarian D, Roger VL, et al. Heart Disease and Stroke Statistics--2014 Update: A Report From the American Heart Association. *Circulation.* Dec 18 2013.
6. Heidenreich PA, Albert NM, Allen LA, et al. Forecasting the impact of heart failure in the United States: a policy statement from the American Heart Association. *Circ. Heart Fail.* May 2013;6(3):606-619.
7. Slaughter MS, Rogers JG, Milano CA, et al. Advanced heart failure treated with continuous-flow left ventricular assist device. *N. Engl. J. Med.* Dec 3 2009;361(23):2241-2251.
8. Hershberger RE, Nauman D, Walker TL, Dutton D, Burgess D. Care processes and clinical outcomes of continuous outpatient support with inotropes (COSI) in patients with refractory endstage heart failure. *J. Card. Fail.* Jun 2003;9(3):180-187.
9. Rose EA, Gelijns AC, Moskowitz AJ, et al. Long-term use of a left ventricular assist device for end-stage heart failure. *N. Engl. J. Med.* Nov 15 2001;345(20):1435-1443.
10. Rogers JG, Butler J, Lansman SL, et al. Chronic mechanical circulatory support for inotrope-dependent heart failure patients who are not transplant candidates: results of the INTrEPID Trial. *J. Am. Coll. Cardiol.* Aug 21 2007;50(8):741-747.
11. Rogers JG, Aaronson KD, Boyle AJ, et al. Continuous flow left ventricular assist device improves functional capacity and quality of life of advanced heart failure patients. *J. Am. Coll. Cardiol.* Apr 27 2010;55(17):1826-1834.
12. Grady KL, Meyer PM, Dressler D, et al. Longitudinal change in quality of life and impact on survival after left ventricular assist device implantation. *Ann. Thorac. Surg.* Apr 2004;77(4):1321-1327.
13. Feldman D, Pamboukian SV, Teuteberg JJ, et al. The 2013 International Society for Heart and Lung Transplantation Guidelines for mechanical circulatory support: executive summary. *J. Heart Lung Transplant.* Feb 2013;32(2):157-187.
14. Flint KM, Matlock DD, Lindenfeld J, Allen LA. Frailty and the selection of patients for destination therapy left ventricular assist device. *Circ. Heart Fail.* Mar 1 2012;5(2):286-293.

15. Baker K, Flattery M, Salyer J, Haugh KH, Maltby M. Caregiving for patients requiring left ventricular assistance device support. *Heart Lung.* May-Jun 2010;39(3):196-200.
16. Magid M, Jones J, Allen LA, McIlvennan CK, Magid K, Thompson JS, Matlock DD. The experience of caregivers of LVAD patients: a meta-synthesis. Paper presented at: University of Colorado School of Medicine 2013 Palliative Care Research Day; October 29, 2013, 2013; Aurora, CO.
17. Bunzel B, Laederach-Hofmann K, Wieselthaler G, Roethy W, Wolner E. Mechanical circulatory support as a bridge to heart transplantation: what remains? Long-term emotional sequelae in patients and spouses. *J. Heart Lung Transplant.* Apr 2007;26(4):384-389.
18. Kitko LA, Hupcey JE, Gilchrist JH, Boehmer JP. Caring for a spouse with end-stage heart failure through implantation of a left ventricular assist device as destination therapy. *Heart Lung.* May-Jun 2013;42(3):195-201.
19. Allen LA, Stevenson LW, Grady KL, et al. Decision making in advanced heart failure: a scientific statement from the American Heart Association. *Circulation.* Apr 17 2012;125(15):1928-1952.
20. Fowler FJ, Jr., Levin CA, Sepucha KR. Informing and involving patients to improve the quality of medical decisions. *Health Aff. (Millwood).* Apr 2011;30(4):699-706.
21. Sepucha KR, Fowler FJ, Jr., Mulley AG, Jr. Policy support for patient-centered care: the need for measurable improvements in decision quality. *Health Aff. (Millwood).* 2004;Suppl Variation:VAR54-62.
22. Sepucha K, Ozanne E, Silvia K, Partridge A, Mulley AG, Jr. An approach to measuring the quality of breast cancer decisions. *Patient Educ. Couns.* Feb 2007;65(2):261-269.
23. Zikmund-Fisher BJ, Couper MP, Singer E, et al. Deficits and variations in patients' experience with making 9 common medical decisions: the DECISIONS survey. *Med. Decis. Making.* Sep-Oct 2010;30(5 Suppl):85S-95S.
24. Elwyn G, O'Connor A, Stacey D, et al. Developing a quality criteria framework for patient decision aids: online international Delphi consensus process. *BMJ.* Aug 26 2006;333(7565):417.
25. Teno JM, Fisher ES, Hamel MB, Coppola K, Dawson NV. Medical care inconsistent with patients' treatment goals: association with 1-year Medicare resource use and survival. *J. Am. Geriatr. Soc.* Mar 2002;50(3):496-500.
26. Karlawish JH, Casarett DJ, James BD, Xie SX, Kim SY. The ability of persons with Alzheimer disease (AD) to make a decision about taking an AD treatment. *Neurology.* May 10 2005;64(9):1514-1519.
27. Gazmararian JA, Baker DW, Williams MV, et al. Health literacy among Medicare enrollees in a managed care organization. *JAMA.* Feb 10 1999;281(6):545-551.
28. Say R, Murtagh M, Thomson R. Patients' preference for involvement in medical decision making: a narrative review. *Patient Educ. Couns.* Feb 2006;60(2):102-114.
29. Iacovetto M, Matlock DD, McIlvennan CK, Thompson JS, Bradley WJ, Allen LA. Existing tools for patients considering a left ventricular assist device: a cross-sectional review of internet, print and multimedia materials. *Submitted to JAMA IM [under review].* 12/23/2013.
30. McIlvennan CK, Narayan MP, Thompson JS, Cannon A, Bradley WJ, Nowels C, Matlock DD, Allen LA. Patient Decision Making and Preparation for Destination Therapy Left Ventricular Assist Devices: A Nationwide Sampling of Mechanical Circulatory Support Coordinators. University of Colorado School of Medicine 2013 Palliative Care Research Day; October 29, 2013, 2013; Aurora, CO.
31. McIlvennan CK, Allen L, Nowels CT, Cleveland JC, Brieke A, Matlock DD. Decision making for destination therapy left ventricular assist devices: "There was no choice" versus "I thought about it an awful lot.". *Submitted to Circ Cardiovasc Outcomes [under review].* 11/13/2013.
32. McIlvennan CK, Magid K, Ambardekar AV, Thompson JS, Matlock DD, Allen LA. Clinical outcomes following continuous-flow left ventricular assist device: a systematic review. *Submitted to JACC [under review].* 12/19/2013.

33. University of Ottawa. Ottawa Decision Support Framework. 2010. http://decisionaid.Ohri.Ca/docs/develop/odsf.Pdf. Accessed June 2, 2012.
34. U.S. Government. The Patient Protection and Affordable Care Act. 2010.
35. Sepucha KR, Levin CA, Uzogara EE, Barry MJ, O'Connor AM, Mulley AG. Developing instruments to measure the quality of decisions: early results for a set of symptom-driven decisions. *Patient Educ. Couns.* Dec 2008;73(3):504-510.
36. Mdege ND, Man MS, Taylor Nee Brown CA, Torgerson DJ. Systematic review of stepped wedge cluster randomized trials shows that design is particularly used to evaluate interventions during routine implementation. *J. Clin. Epidemiol.* Sep 2011;64(9):936-948.
37. Barry MJ, Fowler FJ, Jr., Mulley AG, Jr., Henderson JV, Jr., Wennberg JE. Patient reactions to a program designed to facilitate patient participation in treatment decisions for benign prostatic hyperplasia. *Medical Care*. 1995;33:771-782
38. Sepucha KR, Levin CA, Uzogara EE, Barry MJ, O'Connor AM, Mulley AG. Developing instruments to measure the quality of decisions: early results for a set of symptom-driven decisions. *Patient Educ. Couns.* Dec 2008;73(3):504-510.
39. O'Connor AM. Validation of a decisional conflict scale. *Medical Decision Making.15(1):25-30,*. 1995:Mar
40. Brehaut JC, O'Connor AM, Wood TJ, Hack TF, Siminoff L, Gordon E, Feldman-Stewart D. Validation of a decision regret scale. *Medical Decision Making*. 2003;23:281-292
41. Degner LF, Sloan JA, Venkatesh P. The control preferences scale. *Can J Nurs Res*. 1997; 29:21-43.
42. Cohen S, Kamarck T, Mermelstein R. A global measure of pereceived stress. *Journal of Health and Social Behavior*. 1983; 24, 386-396.
43. Zigmond AS, Snaith RP. The hospital anxiety and depression scale. *Acta Psychatr Scand. 1983*; 67(6): 361-70.
44. Mack JW, Nilsson M, Balboni T, Friedlander RJ, Block SD, Trice E, Prigerson HG. Peace, Equanimity, and Acceptance in the Cancer Experience (PEACE): Validation of a scale to assess acceptance and struggle with terminal illness. *Cancer*. 2008 Jun;112(11):2509-17.
45. EuroQol Group. EuroWol – a new facility for the measurement of health-related quality of life. *Health Policy*. 1990; 16, 119-208.
46. Archbold PG, Stewart BJ, Greenlick MR, Harvath T. Mutuality and preparedness as predictors of caregiver role strain. *Research in Nursing & Health*. 1990; 13, 375-384.
47. Heyland DK, Cook DJ, Rocker GM, Dodek PM, Kutsogiannis DJ, Skrobik Y, Jiang X, Day AG, Cohen SR. The development and validation of a novel questionnaire to measure patient and family satisfaction with end-of-life care: The Canadian Health Care Evaluation Project (CANHELP) Questionnaire. *Palliative Medicine*. 2010; 24(7), 682-95.
48. Heyland DK, Tranmer JE. Measuring family satisfaction with care in the intensive care unit: The development of a questionnaire and preliminary results. *Journal of Critical Care*. December 2001; 16(4), 142-149.
