# Supplementary material for: Effectiveness of an Intervention Supporting Shared Decision Making for Destination Therapy Left Ventricular Assist Device: The DECIDE-LVAD Randomized Clinical Trial
Source: JAMA Intern Med. 2018 Feb 26;178(4):520–9. doi: 10.1001/jamainternmed.2017.8713 (PMC5876922; doi:10.1001/jamainternmed.2017.8713)
Supplement: Supplement 2. — eAppendix 1. Pamphlet. A decision aid for Left Ventricular Assist Device (LVAD) as Destination Therapy eAppendix 2. Sample Surveys. Search strategy for EMBASE (using Embase.com) eAppendix 3. Missing Data. Flow of information through the different phases of the review eTable. Participant Characteristics by Missing Primary Knowledge (at Baseline 1 and Baseline 2) and Values (at 1-Month) Outcomes [file jamainternmed-178-520-s002.pdf]

Supplementary Online Content

Allen AL, McIlvennan CK, Thompson ST, Dunlay SM, et al. Effectiveness of an intervention supporting shared decision making for destination therapy left ventricular assist device: the DECIDE-LVAD randomized clinical trial [published online February 26, 2018]. *JAMA Intern Med*. doi:10.1001/jamainternmed.2017.8713

eAppendix 1. Pamphlet. A decision aid for Left Ventricular Assist Device (LVAD) as Destination Therapy
eAppendix 2. Sample Surveys. Search strategy for EMBASE (using Embase.com)
eAppendix 3. Missing Data. Flow of information through the different phases of the review
eTable. Participant Characteristics by Missing Primary Knowledge (at Baseline 1 and Baseline 2) and Values (at 1-Month) Outcomes

This supplementary material has been provided by the authors to give readers additional information about their work.

© 2018 Allen LA et al. JAMA Internal Medicine.

A decision aid for

Left Ventricular Assist Device (LVAD) as Destination Therapy

A device for patients with advanced heart failure

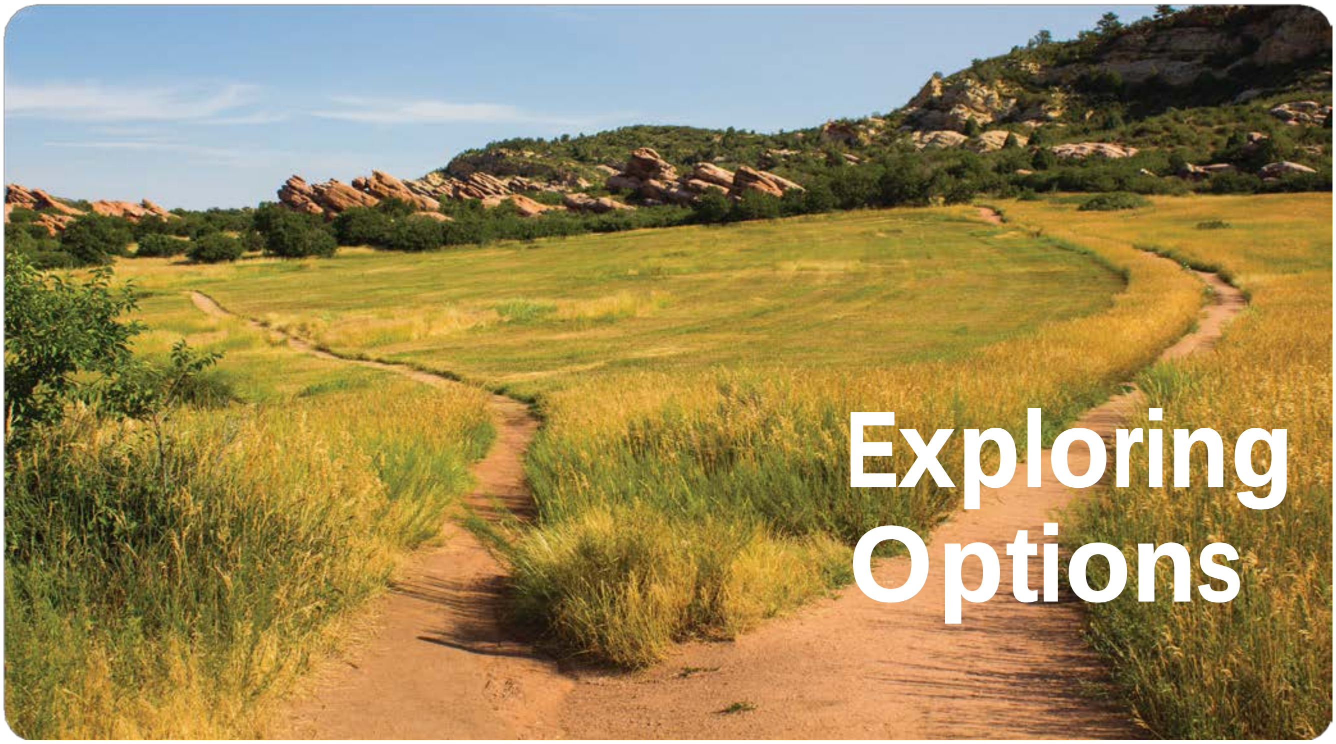

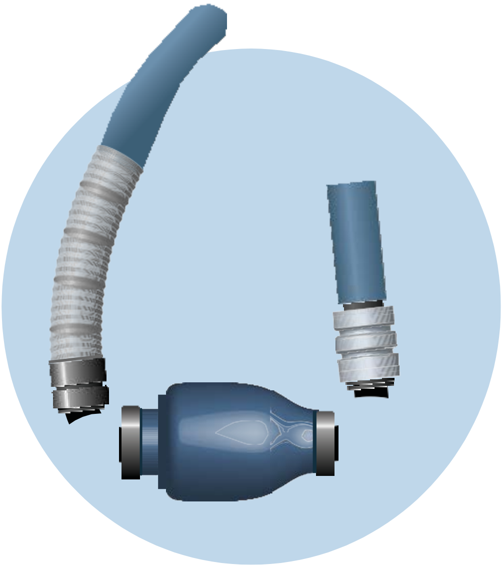

You are being considered for an LVAD. This booklet is designed to help you understand what an LVAD is and to help you, your family, and your doctors think about what is best for you. Your values and goals are the most important factors in making a decision.

What are your current feelings about being considered for an LVAD?

Think about…

- how you want to live the rest of your life
- your hopes and fears
- your biggest questions

© 2018 Allen LA et al. JAMA Internal Medicine.

Now that you've thought about your first impressions, let's talk through the details of this DECISION.

You are in a tough spot.

You have severe heart failure. Your current medicines are no longer working. Without a major heart surgery, you may die soon. You are not able to get a transplant. Therefore, you are being considered for an LVAD (partial artificial heart).

Many patients like you have found this scary or confusing. Some patients have felt pressured to make a decision.

These emotions are normal.

What is heart failure?

Heart failure is when the heart is too weak to pump enough blood for the body. This causes shortness of breath, tiredness, and swelling. For most people, heart failure gets worse over time. Severe heart failure can lead to death.

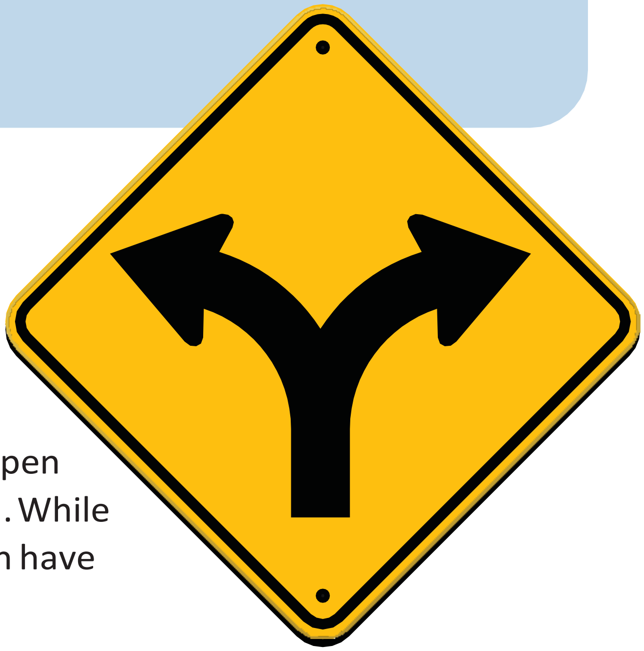

This is a major decision.

If your doctors feel you are eligible for an LVAD, you are facing a major decision. Such a decision may make some people feel uncomfortable. While no one can predict the future, understanding what could happen may help you to feel more at peace about your decision. While this may be hard to think about, people in your position have wanted to know this information.

Some patients choose to get an LVAD. Other patients decide not to get an LVAD. The right choice really depends on how you hope to live the rest of your life.

© 2018 Allen LA et al. JAMA Internal Medicine.

What is an LVAD (Left Ventricular Assist Device)?

An LVAD is a mechanical device that helps your heart do its job of pumping blood. The LVAD is attached to your heart during major surgery. An LVAD in a patient who will never get a transplant is called "destination therapy" (or DT LVAD). This means that the patient will have the device for the rest of his or her life.

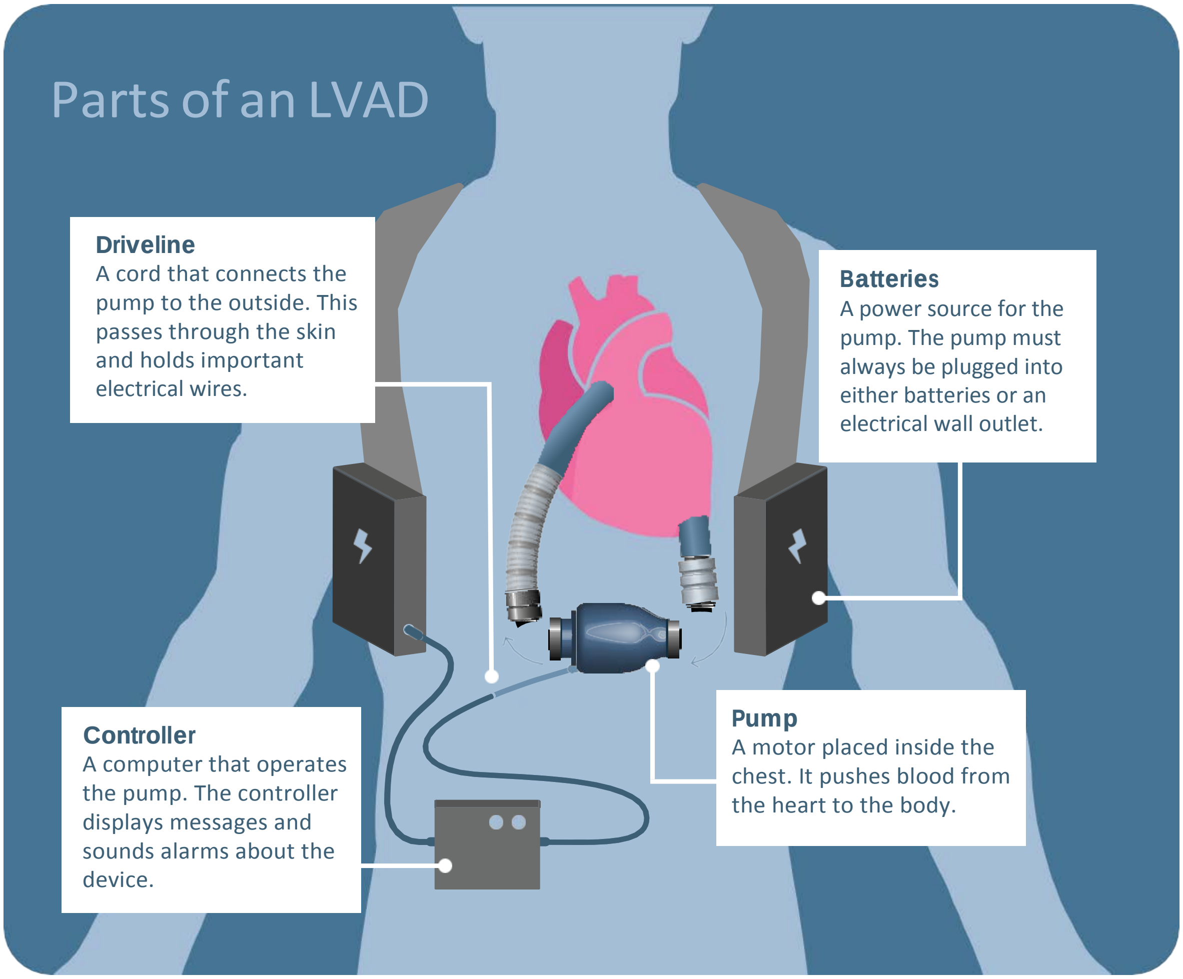

What is the process of getting an LVAD?

- Getting an LVAD requires open heart surgery
- The surgery can be dangerous, and recovery can be quite long and difficult
- Most patients stay in the hospital for a couple of weeks – some stay longer
- Patients and their caregivers attend education sessions to learn how to use the device equipment

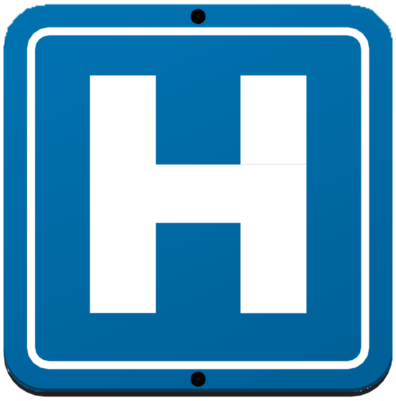

© 2018 Allen LA et al. JAMA Internal Medicine.

What might my life look like with each OPTION?

The numbers below are a summary of information collected from recent medical studies. However, no one can know what will happen to an individual person.

Life *with* an LVAD

How long might I live?

Patients usually live longer with an LVAD. After 1 year, about 8 out of 10 patients who got an LVAD are still alive.[1]

80%

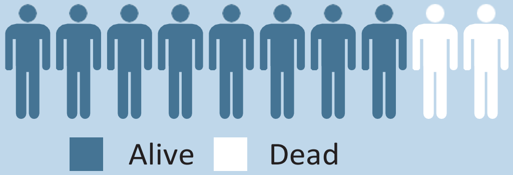

How might I feel?

Of those patients who get through surgery, many feel big improvements in heart failure symptoms – less shortness of breath, less swelling, and more energy.[1] Most patients say they can do more.

What complications might occur?

1 year after surgery, about:[1]

- 5 to 6 patients out of 10 are readmitted to the hospital

55%

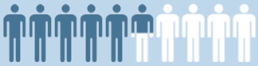

- 1 in 10 have a disabling stroke

10%

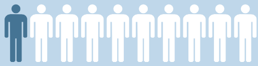

- 2 in 10 develop a device-related infection

20%

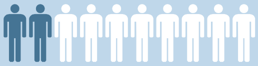

- 2 in 10 have a serious bleed that requires medical attention

20%

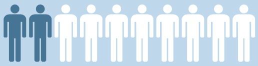

- Nearly 1 in 10 need surgery again to replace the LVAD pump

5%

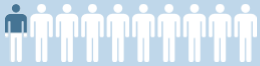

Life *without* an LVAD

How long might I live?

Patients usually do not live as long without an LVAD. After 1 year, almost 2 out of 10 patients who did not get an LVAD are still alive.[1]

17%

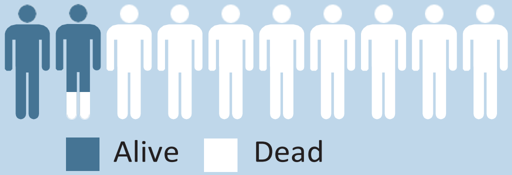

How might I feel?

Nearly all patients without an LVAD continue to have severe heart failure. Symptoms can be managed.

What might occur if I don't get an LVAD?

Most likely, patients will not be alive at 1 year.[1]

- Patients will not have to be dependent on a machine to live
- Patients can often leave the hospital earlier and spend their remaining time at home
- Patients often decide to only take medicine to help with pain and other symptoms
- Palliative care and hospice are available, but without an LVAD, patients might need these services sooner (see description on next page)

© 2018 Allen LA et al. JAMA Internal Medicine.

With or without an LVAD, there are services available to help with SYMPTOMS and suffering of advanced illness.

What is palliative care?

Palliative care is medical care for people with serious illnesses. It helps provide relief from symptoms, pain, and stress. It also provides emotional and spiritual support. The goal of palliative care is to improve quality of life for patients and their caregivers.

What is hospice?

Hospice care is given by health professionals for patients near the end of their lives. This care includes medical, emotional, and spiritual support, and helps to provide comfort and peace for patients. Hospice care usually occurs at a patient's home. It can also occur in other settings, such as a nursing home.

Even with an LVAD, some patients will continue to feel worse or get sicker due to other health problems. These patients will have the option of turning off the pump.

Whether we like it or not, everybody eventually dies.

However, you have some control over how you will live the rest of your life.

Patient Perspectives:

Patients who got an LVAD

"It wasn't an easy choice for me to make. But then I started focusing on my life. On myself."

"I was willing to do anything they told me I had to do to feel better."

Patients who chose not to get an LVAD

"I don't know if the pump would keep me alive. And even if it does, I'm not sure it would be worth living. Because I'm not going to claw and hold on to the wall to stay alive."

"I'm tired of being in pain and suffering. I'm not scared of death."

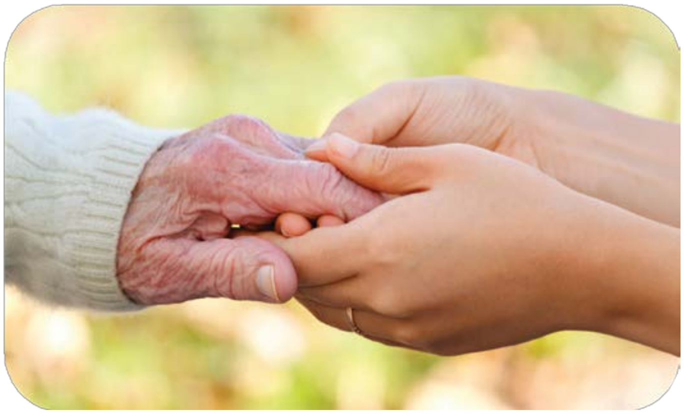

© 2018 Allen LA et al. JAMA Internal Medicine.

If I get an LVAD, how will my life CHANGE?

There are many life-changing aspects of the LVAD that you should consider:

Power Source: You must be plugged into a power source at all times – loss of electrical power to the pump can result in death. When you are sleeping you will plug into an electrical outlet. During the day you can switch over to batteries, which last about 6-12 hours.

Carrying Equipment: Along with batteries, you will have to carry the controller. Equipment can be carried in a vest or on a belt. Battery packs weigh about 7 pounds. Carrying extra battery packs and an extra controller is also important, in case they need to be changed.

Driveline Care: The driveline site (where the electrical cord exits the skin) must be managed carefully. Bandages must be changed and the site should be cleaned several times a week. Lack of care could cause a deadly infection.

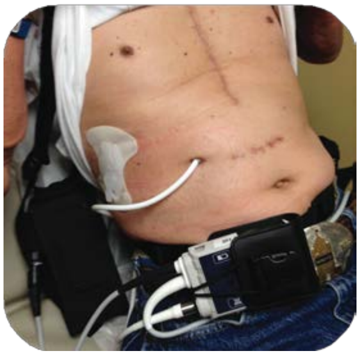

Driveline Site

Medicine: With an LVAD, you will have to take blood thinners for the rest of your life. You may need to continue some medicine to help with your heart, but most patients take less medicine after getting an LVAD. You also will have to go to the doctor's office often for check-ups.

Water Precautions: Due to the equipment and driveline site, you must avoid going into water. This means you can no longer swim, bathe, or take regular showers. You can purchase special water protection equipment in order to shower with your device.

Cost: Depending on your insurance coverage, you may have extra costs with your device. Many patients have to pay for the driveline bandages. You may also have co-payments for your medical care.

Sex: Some patients and their partners report a change in their sex life. However, for most LVAD patients, sex is safe after recovery from surgery.

Emotions: The LVAD may cause emotional effects for both patients and caregivers. Some people are very grateful for the LVAD and get used to these lifestyle changes. Others worry often about their equipment failing or feel overwhelmed with taking care of the device. This stress can lead to depression and anxiety. Seeking mental health care may be helpful.

© 2018 Allen LA et al. JAMA Internal Medicine.

An LVAD is a major decision for CAREGIVERS, too.

Caring for a patient with severe heart failure often requires lifestyle changes. When a patient gets an LVAD, the caregiver's lifestyle can change further. The caregiver's responsibilities are different for every patient and change over time.

Most LVAD caregivers express happiness in being able to help their loved one. However, some caregivers feel stressed with responsibilities, finances, or the health of the patient.

Caregivers of patients who choose not to get an LVAD may also experience similar responsibilities and feelings.

The LVAD caregiver helps with:

- Driveline site bandage changes and checking for infection
- Battery care
- Equipment care and alarm response
- Managing medicines
- Arranging follow-up appointments
- Extra care during recovery, usually the first couple months after surgery:
  - Sponge bathing
  - Cooking
  - Running errands
  - Driving the patient

Things to consider:

- Most hospital programs require LVAD patients have a caregiver
- The primary caregiver usually attends training to learn how to operate the LVAD and care for the patient
- Caregivers must be committed to helping the patient on a daily basis – this usually decreases over time
- Some LVAD programs offer support for caregivers – check with your local program to see what is available for caregivers and families

Caregiver Perspectives:

Caregivers of patients with an LVAD

"I am so thankful for the LVAD. You learn to deal with those little things."

"I've never regretted this decision. It's just that sometimes you get to the point where you want to be at the house and not have someone there that you need to constantly take care of."

Caregivers of patients without an LVAD

"My husband would have been through a lot more stress if he had the LVAD. He died the way he wanted to. He died at home and he died very peacefully. He wasn't in pain and that's brought a lot of peace to me."

"My worries after we made the decision to not get the LVAD were how much worse he was going to get and how long I would be able to keep him at home and look after him."

© 2018 Allen LA et al. JAMA Internal Medicine.

Take some time to CONSIDER what you have learned about LVADs and think about how you want to live the rest of your life.

On a Scale…

How do you want to live out the rest of your life? (check one box)

Do everything I can to live longer, even if that means having major surgery and being dependent on a machine.	☐ ☐ ☐ ☐ ☐ ☐ ☐ ☐ ☐ ☐	Live with whatever time I have left, without going through major surgery or being dependent on a machine.

Reflection…	With an LVAD?	Without an LVAD?
What do you hope for with or without an LVAD?		
What frightens you about living with or without a LVAD?		

What are your biggest questions…

…for your doctor, surgeon, or cardiologist? ____________________

____

…for your caregiver or family? ____________________

____

…for patients who currently have an LVAD? ____________________

____

https://patientdecisionaid.org

1. McIlvennan CK, Magid KH, Ambardekar AV, Thompson JS, Matlock DD, Allen LA. Clinical Outcomes Following Continuous-Flow Left Ventricular Assist Device: A Systematic Review. Circ. Heart Fail. Oct 7 2014.

School of Medicine
UNIVERSITY OF COLORADO
ANSCHUTZ MEDICAL CAMPUS

Copyright © 2014 by The Regents of the University of Colorado on behalf of its employees: Larry A. Allen, MD, MHS; Daniel D. Matlock, MD, MPH; Jocelyn S. Thompson, MA; Colleen K. McIlvennan, DNP, ANP; Amy R. Jenkins, MS.
This work was supported through a Patient-Centered Outcomes Research Institute (PCORI) Program Award (1310-06998), the National Heart, Lung, and Blood Institute (K23HL105896) and the National Institutes on Aging (K23AG040696) of the National Institutes of Health, and University of Colorado Department of Medicine Early Career Scholars Program. Conflicts of Interest: Allen – none; Matlock – none, McIlvennan – none; Thompson - none; Jenkins - none. Reading level: 8.0. Last update: 6/17/2015. Contact: 303-724-7967 or larry.allen@ucdenver.edu.
Some rights reserved. No part of this publication may be used in any commercial development or effort without the express prior written permission of the publisher. No part of this publication may be used in any derivative work without first obtaining permission from the publisher and providing acknowledgement thereof. University of Colorado hereby disclaims all liability associated with the use or adoption of the information provided herein. User shall remain liable for any damages resulting from his reliance on this information. The content is solely the responsibility of the authors and does not necessarily represent the official views of funding agencies (NIH, PCORI) or medical centers. The material provided on this infographic is intended for informational purposes only and is not provided as medical advice. Any individual should consult with his or her own physician before determining whether a left ventricular assist device is right for him or her. This work is licensed under a Creative Commons Attribution, Non-Commercial, No-Derivatives 4.0 International License.

CC BY NC ND

© 2018 Allen LA et al. JAMA Internal Medicine.

Baseline 1 Survey – Patient

Date:_/_____/_____________
m m d d y y y y

Part 1: Written

DT LVAD Decision Quality Knowledge

*Please select the best answer to each of the following questions about Destination Therapy Left Ventricular Assist Device (DT LVAD). Select only **ONE** answer to each question.*

1. When people refer to "destination therapy" (DT) this means:
   - ❑ Patients will have the DT LVAD removed at some future destination time.
   - ❑ The DT LVAD is the only option for the patient.
   - ❑ Patients will have the DT LVAD removed when they reach the destination of a heart transplant.
   - ❑ Patients will probably live the rest of their lives with a DT LVAD.

2. When someone decides to get a DT LVAD, which best describes the surgery?
   - ❑ The LVAD pump is put in through a large blood vessel using a large needle.
   - ❑ The LVAD pump is put in through a small cut in the belly during an operation.
   - ❑ The LVAD pump is connected to the heart through open-heart surgery.
   - ❑ The heart is taken out and the LVAD pump is put in its place through open-heart surgery.
   - ❑ The LVAD pump is put next to the defibrillator in the upper chest.

3. For patients who decide not to get a DT LVAD, the alternative is most likely what?
   - ❑ Declining DT LVAD is not an option.
   - ❑ Undergoing a different procedure to fix the heart.
   - ❑ Being more aggressive about pushing up the doses of beta-blockers.
   - ❑ Changing goals and treatments to be more about quality and less about quantity of life, often aided by palliative and hospice care.

4. What is true about the power needs for a DT LVAD to keep working?
   - ❑ The pump needs its battery changed by a surgery about every 5 years.
   - ❑ The DT LVAD must always be plugged into an electrical outlet.
   - ❑ Nothing, it is magnetic.
   - ❑ The rechargeable batteries need to be changed at least once per week.
   - ❑ Patients can hook up to either an electrical outlet or rechargeable batteries.

5. If 100 people get a DT LVAD, about how many will still be alive after 1 year?
   - ❑ 20
   - ❑ 40
   - ❑ 80
   - ❑ 95

© 2018 Allen LA et al. JAMA Internal Medicine.

6. If 100 people get a DT LVAD, about how many of them will have a stroke in the first year?
   - ☐ 2
   - ☐ 10
   - ☐ 25
   - ☐ 40

7. What action reduces this risk of getting an infection of the DT LVAD?
   - ☐ A person should never take the dressing off the power cord where it enters the body.
   - ☐ Cleaning the power cord site with a special disinfectant at least three times per week.
   - ☐ Pulling the power cord out and washing it every day.
   - ☐ Showering every day.
   - ☐ Soaking the area where the power cord enters the body with soapy water.

8. What medication is generally required with all DT LVADs?
   - ☐ Diuretic such as furosemide (Lasix)
   - ☐ Anticoagulant or "blood thinner", such as warfarin (Coumadin)
   - ☐ ACE inhibitor, such as lisinopril (Prinivil)
   - ☐ Beta-blocker, such as carvedilol (Coreg)
   - ☐ Antidepressant, such as sertraline (Zoloft)

9. Which of the following is most true about a friend or family caregiver for a patient getting a DT LVAD?
   - ☐ A caregiver is needed for the first few days after going home to assist with recovery.
   - ☐ A caregiver is needed to assist with daily activities for at least 3 months after and often longer.
   - ☐ If a patient was able to live alone before the DT LVAD, they do not need a caregiver.
   - ☐ If a patient does not have a friend or family caregiver, the hospital can arrange for home health to do all the caregiver responsibilities.

10. If someone becomes sick and decides that he/she no longer wants to live with the DT LVAD which of the following is the most appropriate option?
    - ☐ The patient can then decide to have a heart transplantation.
    - ☐ The patient usually goes back to the operating room to have the DT LVAD removed.
    - ☐ The patient can ask to have the device turned off, which almost always results in death in less than 20 minutes.
    - ☐ There is no option, the patient must continue living with the DT LVAD.

© 2018 Allen LA et al. JAMA Internal Medicine.

DT LVAD Decision Quality Values

If you were able to choose how to live the rest of your life, between the values at each end of the line below, where do you think you would want to be?

Please mark an “X” on the line where you think you would want to be.

**Do everything I can to live longer**, even if that means having major surgery and being dependent on a machine.	**Live with whatever time I have left,** without going through major surgery or being dependent on a machine.

© 2018 Allen LA et al. JAMA Internal Medicine.

Decision Conflict

O'Connor AM, Decisional Conflict Scale. © 1993 [updated 2005]. www.ohri.ca/decisionaid.

A. Which treatment option do you prefer?
- ❑ Getting a DT LVAD
- ❑ Not getting a DT LVAD
- ❑ Unsure

B. Considering the option you prefer, please answer the following questions:

	Strongly Agree	***Agree***	***Neither Agree Nor Disagree***	***Disagree***	***Strongly Disagree***
1. I know which options are available to me.	❑	❑	❑	❑	❑
2. I know the benefits of each option.	❑	❑	❑	❑	❑
3. I know the risks and side effects of each option.	❑	❑	❑	❑	❑
4. I am clear about which benefits matter most to me.	❑	❑	❑	❑	❑
5. I am clear about which risks and side effects matter most to me.	❑	❑	❑	❑	❑
6. I am clear about which is more important to me (the benefits or the risks and side effects).	❑	❑	❑	❑	❑
7. I have enough support from others to make a choice.	❑	❑	❑	❑	❑
8. I am choosing without pressure from others.	❑	❑	❑	❑	❑
9. I have enough advice to make a choice.	❑	❑	❑	❑	❑
10. I am clear about the best choice for me.	❑	❑	❑	❑	❑
11. I feel sure about what to choose.	❑	❑	❑	❑	❑
12. This decision is easy for me to make.	❑	❑	❑	❑	❑
13. I feel I have made an informed choice.	❑	❑	❑	❑	❑
14. My decision shows what is important to me.	❑	❑	❑	❑	❑
15. I expect to stick with my decision.	❑	❑	❑	❑	❑
16. I am satisfied with my decision.	❑	❑	❑	❑	❑

© 2018 Allen LA et al. JAMA Internal Medicine.

Control Preferences Scale

Degner, Sloan, et al., 1997.

Please select the option that best represents your preferred role in making decisions about your health care:

- ❑ I prefer to make the final selection about which treatment I will receive
- ❑ I prefer to make the final selection of my treatment after seriously considering my doctor's opinion
- ❑ I prefer that my doctor and I share responsibility for deciding which treatment is best for me
- ❑ I prefer that my doctor make the final decision about which treatment will be used, but seriously considers my opinion
- ❑ I prefer to leave all decision regarding treatment to my doctor

© 2018 Allen LA et al. JAMA Internal Medicine.

PEACE Illness Acceptance Scale
American Cancer Society, 2008.

	Not at all	*To a slight extent*	*To some extent*	*To a large extent*
1. To what extent are you able to accept your diagnosis of heart failure?	1	2	3	4
2. To what extent would you say you have a sense of inner peace and harmony?	1	2	3	4
3. To what extent do you feel that you have made peace with your illness?	1	2	3	4
4. Do you feel well loved now?	1	2	3	4
5. To what extent do you feel a sense of inner calm and tranquility?	1	2	3	4
6. To what extent do changes in your physical appearance upset you?	1	2	3	4
7. To what extent does worry about your illness make it difficult for you to live day to day?	1	2	3	4
8. To what extent do you feel like it is unfair for you to have heart failure right now?	1	2	3	4
9. To what extent do you feel like your life, as you know it, is over?	1	2	3	4
10. To what extent do you feel angry about your illness?	1	2	3	4
11.To what extent do you feel that your illness has beaten you down?	1	2	3	4
12. To what extent do you feel ashamed of or embarrassed by your current condition?	1	2	3	4

© 2018 Allen LA et al. JAMA Internal Medicine.

Perceived Stress Scale

American Sociological Association, Cohen S, Kararch T, and Mermelstein R, 1983.

	Never	***Almost Never***	***Sometimes***	***Fairly Often***	***Very Often***
1. In the last month, how often have you been upset because of something that happened unexpectedly?	0	1	2	3	4
2. In the last month, how often have you felt that you were unable to control the important things in your life?	0	1	2	3	4
3. In the last month, how often have you felt nervous and "stressed"?	0	1	2	3	4
4. In the last month, how often have you felt confident about your ability to handle your personal problems?	0	1	2	3	4
5. In the last month, how often have you felt that things were going your way?	0	1	2	3	4
6. In the last month, how often have you found that you could not cope with all the things that you had to do?	0	1	2	3	4
7. In the last month, how often have you been able to control irritations in your life?	0	1	2	3	4
8. In the last month, how often have you felt that you were on top of things?	0	1	2	3	4
9. In the last month, how often have you been angered because of things that were outside of your control?	0	1	2	3	4
10. In the last month, how often have you felt difficulties were piling up so high that you could not overcome them?	0	1	2	3	4

© 2018 Allen LA et al. JAMA Internal Medicine.

The Patient Health Questionnaire-2 (PHQ-2)

Spitzer RL, Williams JBW, Kroenke K. Pfizer Inc.2003.

Use a circle or check mark to indicate your answer.

Over the last two weeks, how often have you been bothered by any of the following problems?

	Not at all	***Several days***	***More than half the days***	***Nearly every day***
1. Little interest or pleasure in doing things.	0	1	2	3
2. Feeling down, depressed, or hopeless.	0	1	2	3

© 2018 Allen LA et al. JAMA Internal Medicine.

Record ID#: _______________

EQ Visual Analogue Scale

1998 EuroQol Group EQ-5D™ is a trade mark of the EuroQol Group.

To help people say how good or bad a health state is, we have drawn a scale (rather like a thermometer) on which the best state you can imagine is marked 100 and the worst state you can imagine is marked 0.

We would like you to indicate on this scale how good or bad your own health is today, in your opinion. Please do this by drawing a line from the box below to whichever point on the scale indicates how good or bad your health state is today.

Your own health state today

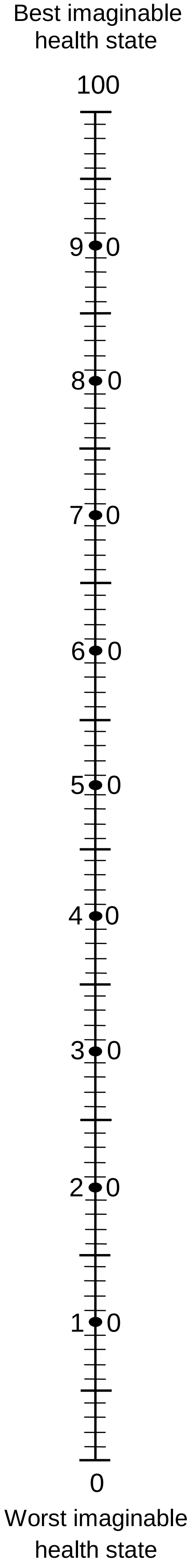

© 2018 Allen LA et al. JAMA Internal Medicine.

Demographics

1. How many years ago did a doctor first tell you that you had a heart problem?
   - ❑ Within the past 2 years
   - ❑ 2 – 4 years
   - ❑ 4 or more
   - ❑ I am not sure

2. Where did you first learn about LVADs as a treatment option?
   - ❑ This hospital
   - ❑ Before coming to this hospital, from a referring provider
   - ❑ Online
   - ❑ Family or friend
   - ❑ Other: ________________________

3. Are you of Hispanic or Latino origin or descent?
   - ❑ Yes, Hispanic or Latino
   - ❑ No, not Hispanic or Latino

4. How would you describe your race? *(Please check only one)*
   - ❑ American Indian or Alaskan Native
   - ❑ Asian or Pacific Islander
   - ❑ Black or African American
   - ❑ White
   - ❑ Another race or multiracial: ________________________

5. What is your current relationship status?
   - ❑ Married
   - ❑ Widowed
   - ❑ Divorced
   - ❑ Single/Never Married
   - ❑ Separated
   - ❑ Member of an Unmarried Couple

6. What was the highest grade you completed in school?
   - ❑ Less than high school graduate
   - ❑ High school graduate or GED certificate
   - ❑ Some college
   - ❑ College graduate
   - ❑ Any post-graduate work

7. Are you currently employed?
   - ❑ Yes – Full time
   - ❑ No – On disability
   - ❑ Yes – Part time
   - ❑ No – Student
   - ❑ No – Retired
   - ❑ No – Homemaker
   - ❑ No – Unemployed
   - ❑ Other: ________________________

8. What is your total household income (your income, plus the income of those you live with, such as your spouse)?
   - ❑ Less than or equal to $20,000
   - ❑ $20,001 – $40,000
   - ❑ $40,001 - $60,000
   - ❑ $60,001 - $80,000
   - ❑ Greater than $80,000

© 2018 Allen LA et al. JAMA Internal Medicine.

Subjective Numeracy Scale

Fagerlin, A., et al. Medical Decision Making, 2007: 27: 672-680.

*For each of the following questions, please check the box that best reflects **how good you are at doing the following things:***

1. How good are you at working with fractions?

❑ 1	❑ 2	❑ 3	❑ 4	❑ 5	❑ 6
**Not at all good**					**Extremely good**

2. How good are you at working with percentages?

❑ 1	❑ 2	❑ 3	❑ 4	❑ 5	❑ 6
**Not at all good**					**Extremely good**

3. How good are you at calculating a 15% tip?

❑ 1	❑ 2	❑ 3	❑ 4	❑ 5	❑ 6
**Not at all good**					**Extremely good**

4. How good are you at figuring out how much a shirt will cost if it is 25% off?

❑ 1	❑ 2	❑ 3	❑ 4	❑ 5	❑ 6
**Not at all good**					**Extremely good**

*For each of the following questions, please check the box that **best reflects your answer:***

5. When reading the newspaper, how **helpful** do you find tables and graphs that are parts of a story?

❑ 1	❑ 2	❑ 3	❑ 4	❑ 5	❑ 6
**Not at all helpful**					**Extremely helpful**

6. When people tell you the chance of something happening, do you prefer that they use **words** (“it rarely happens”) or **numbers** (“there’s a 1% chance”)?

❑ 1	❑ 2	❑ 3	❑ 4	❑ 5	❑ 6
**Always prefer words**					**Always prefer numbers**

7. When you hear a weather forecast, do you prefer predictions using percentages (e.g., “there will be a 20% chance of rain today”) or predictions using only words (e.g., “there is a small chance of rain today”)?

❑ 1	❑ 2	❑ 3	❑ 4	❑ 5	❑ 6
**Always prefer percentages**					**Always prefer words**

8. How often do you find numerical information to be useful?

❑ 1	❑ 2	❑ 3	❑ 4	❑ 5	❑ 6
**Never**					**Very often**

© 2018 Allen LA et al. JAMA Internal Medicine.

Part 2: Oral

Study Coordinator Instructions: ***Complete the following two survey measures orally with the patient.*** *Hold onto Part 2 of the survey and record answers from patient.*

Short Portable Mental Status Questionnaire (SPMSQ)

Pfeiffer, E. Journal of American Geriatrics Society. 1975. 23, 433-41.

Instructions: Ask questions orally and record answers, marking if correct or not. All responses must be given from memory and without reference to calendar, newspaper or other aids.

Prompt: "First, I would like to ask you some general questions. Some of these will be very simple, while others may be more difficult."

Questions	***Correct***	***Incorrect***	***Instructions***
1. What is today's date? ______________	❑	❑	*Correct when month, date and year are all correct.*
2. What is the day of the week? ______________	❑	❑	*Correct when the day is correct.*
3. What is the name of this place?	❑	❑	*Correct if any description of the location is given. "My home", city/town, name of the hospital/institution.*
4. What is your phone number? ______________	❑	❑	*Correct when the number can be verified or the subject can repeat the same number at a later time in the interview.*
5. How old are you? ______________	❑	❑	*Correct when the stated age corresponds to the date of birth.*
6. When were you born? ______________	❑	❑	*Correct only when the month, date and year are correct.*
7. Who is the president of the US now? ______________	❑	❑	***Answer: Obama.*** *Requires only the correct last name.*
8. Who was the president before him? ______________	❑	❑	***Answer: Bush.*** *Requires only the correct last name.*
9. What was your mother's maiden name? ______________	❑	❑	*Needs no verification. Only requires a female first name plus a last name other than the subject's.*
10. Can you count backward from 20 by 3's? ______________	❑	❑	***Answer: 17, 14, 11, 8, 5, 2.*** *The entire series must be performed correctly to be scored as correct. Any error in the series is scored as incorrect.*

Total number in<u>in</u>correct: ________

Scoring: 1 point per ***incorrect*** *answer and add together, range*

0-10

© 2018 Allen LA et al. JAMA Internal Medicine.

Literacy Assessment (REALM-R) Score Sheet
Bass PF, Wilson JF, Griffith CH. JGIM 2003. 18(12):1036-38.

Instructions: ***Give the person the list of the REALM-R words on the next page.*** *Point to the first word and ask the person to read the 11 words out loud. Be sensitive to dialect, accent, and articulation problems. The words "read" and "test" should be avoided when introducing and administering the REALM-R.*

Prompt: "Sometimes in the health care system, medical words are used that many people are not familiar with. I would like to get an idea of what medical words you are familiar with. Please say these words for me."

Instructions: If the person takes more than five seconds on a word, they should be encouraged to move on to the next word (e.g., say "Let's try the next word."*) If the person begins to miss every word or appears to be struggling or frustrated, tell them,* "Just look down the list and say the words you know."

Scoring: Count as an error any word that is not attempted or is mispronounced. Place a check mark ("√") next to each word the person pronounces correctly, and an "X" next to each word the person does not attempt or mispronounces. "Fat", "Flu" and "Pill" are not scored.

Fat	
Flu	
Pill	
Allergic	________
Jaundice	________
Anemia	________
Fatigue	________
Directed	________
Colitis	________
Constipation	________
Osteoporosis	________

Score:_____out of 8 correct

© 2018 Allen LA et al. JAMA Internal Medicine.

Fat

Flu

Pill

Allergic

Jaundice

Anemia

Fatigue

Directed

Colitis

Constipation

Osteoporosis

© 2018 Allen LA et al. JAMA Internal Medicine.

Baseline 2 Survey – Patient

Date:_/______/______________
m m d d y y y y

DT LVAD Decision Quality Knowledge

*Please select the best answer to each of the following questions about Destination Therapy Left Ventricular Assist Device (DT LVAD). Select only **ONE** answer to each question.*

1. When people refer to "destination therapy" (DT) this means:
   - ❑ Patients will have the DT LVAD removed at some future destination time.
   - ❑ The DT LVAD is the only option for the patient.
   - ❑ Patients will have the DT LVAD removed when they reach the destination of a heart transplant.
   - ❑ Patients will probably live the rest of their lives with a DT LVAD.

2. When someone decides to get a DT LVAD, which best describes the surgery?
   - ❑ The LVAD pump is put in through a large blood vessel using a large needle.
   - ❑ The LVAD pump is put in through a small cut in the belly during an operation.
   - ❑ The LVAD pump is connected to the heart through open-heart surgery.
   - ❑ The heart is taken out and the LVAD pump is put in its place through open-heart surgery.
   - ❑ The LVAD pump is put next to the defibrillator in the upper chest.

3. For patients who decide not to get a DT LVAD, the alternative is most likely what?
   - ❑ Declining DT LVAD is not an option.
   - ❑ Undergoing a different procedure to fix the heart.
   - ❑ Being more aggressive about pushing up the doses of beta-blockers.
   - ❑ Changing goals and treatments to be more about quality and less about quantity of life, often aided by palliative and hospice care.

4. What is true about the power needs for a DT LVAD to keep working?
   - ❑ The pump needs its battery changed by a surgery about every 5 years.
   - ❑ The DT LVAD must always be plugged into an electrical outlet.
   - ❑ Nothing, it is magnetic.
   - ❑ The rechargeable batteries need to be changed at least once per week.
   - ❑ Patients can hook up to either an electrical outlet or rechargeable batteries.

5. If 100 people get a DT LVAD, about how many will still be alive after 1 year?
   - ❑ 20
   - ❑ 40
   - ❑ 80
   - ❑ 95

© 2018 Allen LA et al. JAMA Internal Medicine.

6. If <u>100</u> people get a DT LVAD, about how many of them will have a stroke in the <u>first year</u>?
   - ❑ 2
   - ❑ 10
   - ❑ 25
   - ❑ 40

7. What action reduces this risk of getting an infection of the DT LVAD?
   - ❑ A person should never take the dressing off the power cord where it enters the body.
   - ❑ Cleaning the power cord site with a special disinfectant at least three times per week.
   - ❑ Pulling the power cord out and washing it every day.
   - ❑ Showering every day.
   - ❑ Soaking the area where the power cord enters the body with soapy water.

8. What medication is generally required with all DT LVADs?
   - ❑ Diuretic such as furosemide (Lasix)
   - ❑ Anticoagulant or “blood thinner”, such as warfarin (Coumadin)
   - ❑ ACE inhibitor, such as lisinopril (Prinivil)
   - ❑ Beta-blocker, such as carvedilol (Coreg)
   - ❑ Antidepressant, such as sertraline (Zoloft)

9. Which of the following is most true about a friend or family caregiver for a patient getting a DT LVAD?
   - ❑ A caregiver is needed for the first few days after going home to assist with recovery.
   - ❑ A caregiver is needed to assist with daily activities for at least 3 months after and often longer.
   - ❑ If a patient was able to live alone before the DT LVAD, they do not need a caregiver.
   - ❑ If a patient does not have a friend or family caregiver, the hospital can arrange for home health to do all the caregiver responsibilities.

10. If someone becomes sick and decides that he/she no longer wants to live with the DT LVAD which of the following is the most appropriate option?
    - ❑ The patient can then decide to have a heart transplantation.
    - ❑ The patient usually goes back to the operating room to have the DT LVAD removed.
    - ❑ The patient can ask to have the device turned off, which almost always results in death in less than 20 minutes.
    - ❑ There is no option, the patient must continue living with the DT LVAD.

© 2018 Allen LA et al. JAMA Internal Medicine.

Decision Conflict

O'Connor AM, Decisional Conflict Scale. © 1993 [updated 2005]. www.ohri.ca/decisionaid.

A. Which treatment option do you prefer?
- ❑ Getting a DT LVAD
- ❑ Not getting a DT LVAD
- ❑ Unsure

B. Considering the option you prefer, please answer the following questions:

	Strongly Agree	***Agree***	***Neither Agree Nor Disagree***	***Disagree***	***Strongly Disagree***
1. I know which options are available to me.	❑	❑	❑	❑	❑
2. I know the benefits of each option.	❑	❑	❑	❑	❑
3. I know the risks and side effects of each option.	❑	❑	❑	❑	❑
4. I am clear about which benefits matter most to me.	❑	❑	❑	❑	❑
5. I am clear about which risks and side effects matter most to me.	❑	❑	❑	❑	❑
6. I am clear about which is more important to me (the benefits or the risks and side effects).	❑	❑	❑	❑	❑
7. I have enough support from others to make a choice.	❑	❑	❑	❑	❑
8. I am choosing without pressure from others.	❑	❑	❑	❑	❑
9. I have enough advice to make a choice.	❑	❑	❑	❑	❑
10. I am clear about the best choice for me.	❑	❑	❑	❑	❑
11. I feel sure about what to choose.	❑	❑	❑	❑	❑
12. This decision is easy for me to make.	❑	❑	❑	❑	❑
13. I feel I have made an informed choice.	❑	❑	❑	❑	❑
14. My decision shows what is important to me.	❑	❑	❑	❑	❑
15. I expect to stick with my decision.	❑	❑	❑	❑	❑
16. I am satisfied with my decision.	❑	❑	❑	❑	❑

© 2018 Allen LA et al. JAMA Internal Medicine.

Acceptability Questionnaire
Adapted from Barry MJ, et al. Med Care 1995, Decision Aid Acceptability.

We would like to know what you think about the LVAD educational materials you recently reviewed.

1. How would you rate the amount of information in the educational materials?
   - ❑ Much less than I needed
   - ❑ A little less than I needed
   - ❑ About the right amount of information
   - ❑ A little more information than I needed
   - ❑ A lot more information than I needed

2. How balanced was the information about getting a DT LVAD versus the other options?
   - ❑ Clearly slanted towards accepting a DT LVAD
   - ❑ A little slanted towards accepting a DT LVAD
   - ❑ Completely balanced
   - ❑ A little slanted towards declining a DT LVAD
   - ❑ Clearly slanted towards declining a DT LVAD

3. Did the educational materials present one option as the best overall choice?
   - ❑ No, the educational materials were neutral and balanced
   - ❑ Yes, the educational materials favored accepting a DT LVAD
   - ❑ Yes, the educational materials favored declining a DT LVAD
   - ❑ Yes, the educational materials favored another option: ________________________

4. How helpful were the educational materials in helping you make a decision about treatment options?
   - ❑ Very helpful
   - ❑ Somewhat helpful
   - ❑ A little helpful
   - ❑ Not helpful

5. Would you recommend the educational materials to other people who are facing the same decision?
   - ❑ I would definitely recommend them
   - ❑ I would probably recommend them
   - ❑ I would probably not recommend them
   - ❑ I would definitely not recommend them

© 2018 Allen LA et al. JAMA Internal Medicine.

1-Month Follow-Up – Patient

Date:_/_____/_____________
m m d d y y y y

DT LVAD Decision Quality Knowledge

*Please select the best answer to each of the following questions about Destination Therapy Left Ventricular Assist Device (DT LVAD). Select only **ONE** answer to each question.*

1. When people refer to “destination therapy” (DT) this means:
   - ❑ Patients will have the DT LVAD removed at some future destination time.
   - ❑ The DT LVAD is the only option for the patient.
   - ❑ Patients will have the DT LVAD removed when they reach the destination of a heart transplant.
   - ❑ Patients will probably live the rest of their lives with a DT LVAD.

2. When someone decides to get a DT LVAD, which best describes the surgery?
   - ❑ The LVAD pump is put in through a large blood vessel using a large needle.
   - ❑ The LVAD pump is put in through a small cut in the belly during an operation.
   - ❑ The LVAD pump is connected to the heart through open-heart surgery.
   - ❑ The heart is taken out and the LVAD pump is put in its place through open-heart surgery.
   - ❑ The LVAD pump is put next to the defibrillator in the upper chest.

3. For patients who decide not to get a DT LVAD, the alternative is most likely what?
   - ❑ Declining DT LVAD is not an option.
   - ❑ Undergoing a different procedure to fix the heart.
   - ❑ Being more aggressive about pushing up the doses of beta-blockers.
   - ❑ Changing goals and treatments to be more about quality and less about quantity of life, often aided by palliative and hospice care.

4. What is true about the power needs for a DT LVAD to keep working?
   - ❑ The pump needs its battery changed by a surgery about every 5 years.
   - ❑ The DT LVAD must always be plugged into an electrical outlet.
   - ❑ Nothing, it is magnetic.
   - ❑ The rechargeable batteries need to be changed at least once per week.
   - ❑ Patients can hook up to either an electrical outlet or rechargeable batteries.

5. If 100 people get a DT LVAD, about how many will still be alive after 1 year?
   - ❑ 20
   - ❑ 40
   - ❑ 80
   - ❑ 95

© 2018 Allen LA et al. JAMA Internal Medicine.

6. If <u>100</u> people get a DT LVAD, about how many of them will have a stroke in the <u>first year</u>?
   - ☐ 2
   - ☐ 10
   - ☐ 25
   - ☐ 40

7. What action reduces this risk of getting an infection of the DT LVAD?
   - ☐ A person should never take the dressing off the power cord where it enters the body.
   - ☐ Cleaning the power cord site with a special disinfectant at least three times per week.
   - ☐ Pulling the power cord out and washing it every day.
   - ☐ Showering every day.
   - ☐ Soaking the area where the power cord enters the body with soapy water.

8. What medication is generally required with all DT LVADs?
   - ☐ Diuretic such as furosemide (Lasix)
   - ☐ Anticoagulant or "blood thinner", such as warfarin (Coumadin)
   - ☐ ACE inhibitor, such as lisinopril (Prinivil)
   - ☐ Beta-blocker, such as carvedilol (Coreg)
   - ☐ Antidepressant, such as sertraline (Zoloft)

9. Which of the following is most true about a friend or family caregiver for a patient getting a DT LVAD?
   - ☐ A caregiver is needed for the first few days after going home to assist with recovery.
   - ☐ A caregiver is needed to assist with daily activities for at least 3 months after and often longer.
   - ☐ If a patient was able to live alone before the DT LVAD, they do not need a caregiver.
   - ☐ If a patient does not have a friend or family caregiver, the hospital can arrange for home health to do all the caregiver responsibilities.

10. If someone becomes sick and decides that he/she no longer wants to live with the DT LVAD which of the following is the most appropriate option?
   - ☐ The patient can then decide to have a heart transplantation.
   - ☐ The patient usually goes back to the operating room to have the DT LVAD removed.
   - ☐ The patient can ask to have the device turned off, which almost always results in death in less than 20 minutes.
   - ☐ There is no option, the patient must continue living with the DT LVAD.

© 2018 Allen LA et al. JAMA Internal Medicine.

DT LVAD Decision Quality Values

If you were able to choose how to live the rest of your life, between the values at each end of the line below, where do you think you would want to be?

Please mark an "X" on the line where you think you would want to be.

**Do everything I can to live longer**, even if that means having major surgery and being dependent on a machine.	**Live with whatever time I have left,** without going through major surgery or being dependent on a machine.

© 2018 Allen LA et al. JAMA Internal Medicine.

Decision Conflict

O'Connor AM, Decisional Conflict Scale. © 1993 [updated 2005]. www.ohri.ca/decisionaid.

A. Which treatment option do you prefer?
- ❑ Getting a DT LVAD
- ❑ Not getting a DT LVAD
- ❑ Unsure

B. Considering the option you prefer, please answer the following questions:

	Strongly Agree	***Agree***	***Neither Agree Nor Disagree***	***Disagree***	***Strongly Disagree***
1. I know which options are available to me.	❑	❑	❑	❑	❑
2. I know the benefits of each option.	❑	❑	❑	❑	❑
3. I know the risks and side effects of each option.	❑	❑	❑	❑	❑
4. I am clear about which benefits matter most to me.	❑	❑	❑	❑	❑
5. I am clear about which risks and side effects matter most to me.	❑	❑	❑	❑	❑
6. I am clear about which is more important to me (the benefits or the risks and side effects).	❑	❑	❑	❑	❑
7. I have enough support from others to make a choice.	❑	❑	❑	❑	❑
8. I am choosing without pressure from others.	❑	❑	❑	❑	❑
9. I have enough advice to make a choice.	❑	❑	❑	❑	❑
10. I am clear about the best choice for me.	❑	❑	❑	❑	❑
11. I feel sure about what to choose.	❑	❑	❑	❑	❑
12. This decision is easy for me to make.	❑	❑	❑	❑	❑
13. I feel I have made an informed choice.	❑	❑	❑	❑	❑
14. My decision shows what is important to me.	❑	❑	❑	❑	❑
15. I expect to stick with my decision.	❑	❑	❑	❑	❑
16. I am satisfied with my decision.	❑	❑	❑	❑	❑

© 2018 Allen LA et al. JAMA Internal Medicine.

Decision Choice

1. What decision has been made about DT LVAD at this time? *(Please check only one)*
   - ❑ I wanted the DT LVAD and decided to get it
   - ❑ I first wanted the DT LVAD but then decided not to get it
   - ❑ I decided not to get the DT LVAD
   - ❑ I first decided not to get the DT LVAD but then decided I wanted it
   - ❑ The medical team decided I was not eligible for a DT LVAD
   - ❑ The medical team's evaluation of me is not yet finished
   - ❑ I have not yet decided

2. Who is involved in the caregiver plan for you? *(Please check all that apply)*

❑ Wife	❑ Son	❑ Father
❑ Husband	❑ Sister	❑ Friend
❑ Partner	❑ Brother	❑ Other family: ____________________
❑ Daughter	❑ Mother	❑ Other: ________________________

© 2018 Allen LA et al. JAMA Internal Medicine.

Decision Regret

O'Connor AM, 1996, University of Ottawa.

If a decision has not been made about DT LVAD at this time, skip the following 5 questions of the "Decision Regret" measure.

Reflect on the decision made about whether or not to have Destination Therapy Left Ventricular Assist Device (DT LVAD). Show how strongly you agree or disagree with these statements by circling the number that best fits your views about the decision.

	Strongly Agree	***Agree***	***Neither Agree Nor Disagree***	***Disagree***	***Strongly Disagree***
1. It was the right decision	1	2	3	4	5
2. I regret the choice that was made	1	2	3	4	5
3. I would go for the same choice if I had it to do over again	1	2	3	4	5
4. The choice did me a lot of harm	1	2	3	4	5
5. The decision was a wise one	1	2	3	4	5

© 2018 Allen LA et al. JAMA Internal Medicine.

Control Preferences Scale
Degner, Sloan, et al., 1997.

Please select the option that best represents your <u>preferred</u> role in making decisions about your health care:

- ❑ I prefer to make the final selection about which treatment I will receive
- ❑ I prefer to make the final selection of my treatment after seriously considering my doctor's opinion
- ❑ I prefer that my doctor and I share responsibility for deciding which treatment is best for me
- ❑ I prefer that my doctor make the final decision about which treatment will be used, but seriously considers my opinion
- ❑ I prefer to leave all decision regarding treatment to my doctor

Please select the option that best represents your <u>actual</u> role in making decisions about your health care:

- ❑ I made the final decision about which treatment I would receive
- ❑ I made the final selection of my treatment after seriously considering my doctor's opinion
- ❑ My doctor and I shared responsibility for deciding which treatment was best for me
- ❑ My doctor made the final decision about which treatment would be used but seriously considered my opinion
- ❑ My doctor made all the decisions regarding my treatment

© 2018 Allen LA et al. JAMA Internal Medicine.

PEACE Illness Acceptance Scale
American Cancer Society, 2008.

	Not at all	***To a slight extent***	***To some extent***	***To a large extent***
1. To what extent are you able to accept your diagnosis of heart failure?	1	2	3	4
2. To what extent would you say you have a sense of inner peace and harmony?	1	2	3	4
3. To what extent do you feel that you have made peace with your illness?	1	2	3	4
4. Do you feel well loved now?	1	2	3	4
5. To what extent do you feel a sense of inner calm and tranquility?	1	2	3	4
6. To what extent do changes in your physical appearance upset you?	1	2	3	4
7. To what extent does worry about your illness make it difficult for you to live day to day?	1	2	3	4
8. To what extent do you feel like it is unfair for you to have heart failure right now?	1	2	3	4
9. To what extent do you feel like your life, as you know it, is over?	1	2	3	4
10. To what extent do you feel angry about your illness?	1	2	3	4
11.To what extent do you feel that your illness has beaten you down?	1	2	3	4
12. To what extent do you feel ashamed of or embarrassed by your current condition?	1	2	3	4

© 2018 Allen LA et al. JAMA Internal Medicine.

The Patient Health Questionnaire-2 (PHQ-2)

Spitzer RL, Williams JBW, Kroenke K. Pfizer Inc.2003.

Use a circle or check mark to indicate your answer.

Over the last two weeks, how often have you been bothered by any of the following problems?

	Not at all	***Several days***	***More than half the days***	***Nearly every day***
1. Little interest or pleasure in doing things.	0	1	2	3
2. Feeling down, depressed, or hopeless.	0	1	2	3

© 2018 Allen LA et al. JAMA Internal Medicine.

EQ Visual Analogue Scale

1998 EuroQol Group EQ-5D™ is a trade mark of the EuroQol Group.

To help people say how good or bad a health state is, we have drawn a scale (rather like a thermometer) on which the best state you can imagine is marked 100 and the worst state you can imagine is marked 0.

We would like you to indicate on this scale how good or bad your own health is today, in your opinion. Please do this by drawing a line from the box below to whichever point on the scale indicates how good or bad your health state is today.

Your own health state today

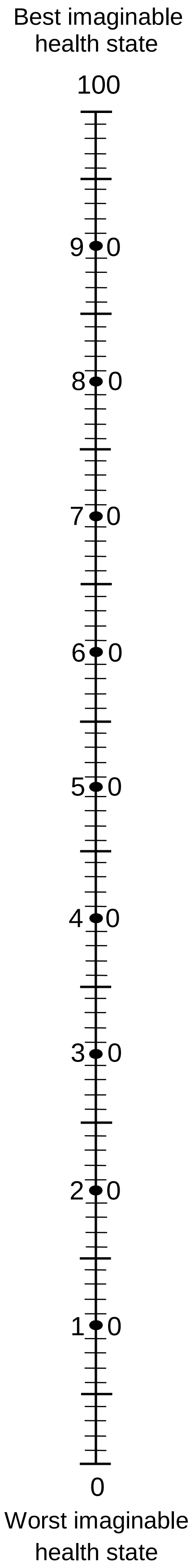

© 2018 Allen LA et al. JAMA Internal Medicine.

6-Month Follow-Up – Patient

Date:_/_____/_____________
m m d d y y y y

DT LVAD Decision Quality Knowledge

*Please select the best answer to each of the following questions about Destination Therapy Left Ventricular Assist Device (DT LVAD). Select only **ONE** answer to each question.*

1. When people refer to “destination therapy” (DT) this means:
   - ❑ Patients will have the DT LVAD removed at some future destination time.
   - ❑ The DT LVAD is the only option for the patient.
   - ❑ Patients will have the DT LVAD removed when they reach the destination of a heart transplant.
   - ❑ Patients will probably live the rest of their lives with a DT LVAD.

2. When someone decides to get a DT LVAD, which best describes the surgery?
   - ❑ The LVAD pump is put in through a large blood vessel using a large needle.
   - ❑ The LVAD pump is put in through a small cut in the belly during an operation.
   - ❑ The LVAD pump is connected to the heart through open-heart surgery.
   - ❑ The heart is taken out and the LVAD pump is put in its place through open-heart surgery.
   - ❑ The LVAD pump is put next to the defibrillator in the upper chest.

3. For patients who decide not to get a DT LVAD, the alternative is most likely what?
   - ❑ Declining DT LVAD is not an option.
   - ❑ Undergoing a different procedure to fix the heart.
   - ❑ Being more aggressive about pushing up the doses of beta-blockers.
   - ❑ Changing goals and treatments to be more about quality and less about quantity of life, often aided by palliative and hospice care.

4. What is true about the power needs for a DT LVAD to keep working?
   - ❑ The pump needs its battery changed by a surgery about every 5 years.
   - ❑ The DT LVAD must always be plugged into an electrical outlet.
   - ❑ Nothing, it is magnetic.
   - ❑ The rechargeable batteries need to be changed at least once per week.
   - ❑ Patients can hook up to either an electrical outlet or rechargeable batteries.

5. If 100 people get a DT LVAD, about how many will still be alive after 1 year?
   - ❑ 20
   - ❑ 40
   - ❑ 80
   - ❑ 95

© 2018 Allen LA et al. JAMA Internal Medicine.

6. If <u>100</u> people get a DT LVAD, about how many of them will have a stroke in the <u>first year</u>?
   - ❑ 2
   - ❑ 10
   - ❑ 25
   - ❑ 40

7. What action reduces this risk of getting an infection of the DT LVAD?
   - ❑ A person should never take the dressing off the power cord where it enters the body.
   - ❑ Cleaning the power cord site with a special disinfectant at least three times per week.
   - ❑ Pulling the power cord out and washing it every day.
   - ❑ Showering every day.
   - ❑ Soaking the area where the power cord enters the body with soapy water.

8. What medication is generally required with all DT LVADs?
   - ❑ Diuretic such as furosemide (Lasix)
   - ❑ Anticoagulant or “blood thinner”, such as warfarin (Coumadin)
   - ❑ ACE inhibitor, such as lisinopril (Prinivil)
   - ❑ Beta-blocker, such as carvedilol (Coreg)
   - ❑ Antidepressant, such as sertraline (Zoloft)

9. Which of the following is most true about a friend or family caregiver for a patient getting a DT LVAD?
   - ❑ A caregiver is needed for the first few days after going home to assist with recovery.
   - ❑ A caregiver is needed to assist with daily activities for at least 3 months after and often longer.
   - ❑ If a patient was able to live alone before the DT LVAD, they do not need a caregiver.
   - ❑ If a patient does not have a friend or family caregiver, the hospital can arrange for home health to do all the caregiver responsibilities.

10. If someone becomes sick and decides that he/she no longer wants to live with the DT LVAD which of the following is the most appropriate option?
   - ❑ The patient can then decide to have a heart transplantation.
   - ❑ The patient usually goes back to the operating room to have the DT LVAD removed.
   - ❑ The patient can ask to have the device turned off, which almost always results in death in less than 20 minutes.
   - ❑ There is no option, the patient must continue living with the DT LVAD.

© 2018 Allen LA et al. JAMA Internal Medicine.

DT LVAD Decision Quality Values

If you were able to choose how to live the rest of your life, between the values at each end of the line below, where do you think you would want to be?

Please mark an "X" on the line where you think you would want to be.

**Do everything I can to live longer**, even if that means having major surgery and being dependent on a machine.	**Live with whatever time I have left,** without going through major surgery or being dependent on a machine.

© 2018 Allen LA et al. JAMA Internal Medicine.

Decision Conflict

O'Connor AM, Decisional Conflict Scale. © 1993 [updated 2005]. www.ohri.ca/decisionaid.

A. Which treatment option do you prefer?
- ❑ Getting a DT LVAD
- ❑ Not getting a DT LVAD
- ❑ Unsure

B. Considering the option you prefer, please answer the following questions:

	Strongly Agree	***Agree***	***Neither Agree Nor Disagree***	***Disagree***	***Strongly Disagree***
1. I know which options are available to me.	❑	❑	❑	❑	❑
2. I know the benefits of each option.	❑	❑	❑	❑	❑
3. I know the risks and side effects of each option.	❑	❑	❑	❑	❑
4. I am clear about which benefits matter most to me.	❑	❑	❑	❑	❑
5. I am clear about which risks and side effects matter most to me.	❑	❑	❑	❑	❑
6. I am clear about which is more important to me (the benefits or the risks and side effects).	❑	❑	❑	❑	❑
7. I have enough support from others to make a choice.	❑	❑	❑	❑	❑
8. I am choosing without pressure from others.	❑	❑	❑	❑	❑
9. I have enough advice to make a choice.	❑	❑	❑	❑	❑
10. I am clear about the best choice for me.	❑	❑	❑	❑	❑
11. I feel sure about what to choose.	❑	❑	❑	❑	❑
12. This decision is easy for me to make.	❑	❑	❑	❑	❑
13. I feel I have made an informed choice.	❑	❑	❑	❑	❑
14. My decision shows what is important to me.	❑	❑	❑	❑	❑
15. I expect to stick with my decision.	❑	❑	❑	❑	❑
16. I am satisfied with my decision.	❑	❑	❑	❑	❑

© 2018 Allen LA et al. JAMA Internal Medicine.

Decision Choice

1. What decision has been made about DT LVAD at this time? *(Please check only one)*
   - ❑ I wanted the DT LVAD and decided to get it
   - ❑ I first wanted the DT LVAD but then decided not to get it
   - ❑ I decided not to get the DT LVAD
   - ❑ I first decided not to get the DT LVAD but then decided I wanted it
   - ❑ The medical team decided I was not eligible for a DT LVAD
   - ❑ The medical team's evaluation of me is not yet finished
   - ❑ I have not yet decided

2. Who is involved in the caregiver plan for you? *(Please check all that apply)*

❑ Wife	❑ Son	❑ Father
❑ Husband	❑ Sister	❑ Friend
❑ Partner	❑ Brother	❑ Other family: ____________________
❑ Daughter	❑ Mother	❑ Other: ________________________

© 2018 Allen LA et al. JAMA Internal Medicine.

Decision Regret

O'Connor AM, 1996, University of Ottawa.

If a decision has not been made about DT LVAD at this time, skip the following 5 questions of the "Decision Regret" measure.

Reflect on the decision made about whether or not to have Destination Therapy Left Ventricular Assist Device (DT LVAD). Show how strongly you agree or disagree with these statements by circling the number that best fits your views about the decision.

	Strongly Agree	***Agree***	***Neither Agree Nor Disagree***	***Disagree***	***Strongly Disagree***
1. It was the right decision	1	2	3	4	5
2. I regret the choice that was made	1	2	3	4	5
3. I would go for the same choice if I had it to do over again	1	2	3	4	5
4. The choice did me a lot of harm	1	2	3	4	5
5. The decision was a wise one	1	2	3	4	5

© 2018 Allen LA et al. JAMA Internal Medicine.

Control Preferences Scale
Degner, Sloan, et al., 1997.

Please select the option that best represents your preferred role in making decisions about your health care:

- ❑ I prefer to make the final selection about which treatment I will receive
- ❑ I prefer to make the final selection of my treatment after seriously considering my doctor's opinion
- ❑ I prefer that my doctor and I share responsibility for deciding which treatment is best for me
- ❑ I prefer that my doctor make the final decision about which treatment will be used, but seriously considers my opinion
- ❑ I prefer to leave all decision regarding treatment to my doctor

Please select the option that best represents your actual role in making decisions about your health care:

- ❑ I made the final decision about which treatment I would receive
- ❑ I made the final selection of my treatment after seriously considering my doctor's opinion
- ❑ My doctor and I shared responsibility for deciding which treatment was best for me
- ❑ My doctor made the final decision about which treatment would be used but seriously considered my opinion
- ❑ My doctor made all the decisions regarding my treatment

© 2018 Allen LA et al. JAMA Internal Medicine.

PEACE Illness Acceptance Scale
American Cancer Society, 2008.

	Not at all	***To a slight extent***	***To some extent***	***To a large extent***
1. To what extent are you able to accept your diagnosis of heart failure?	1	2	3	4
2. To what extent would you say you have a sense of inner peace and harmony?	1	2	3	4
3. To what extent do you feel that you have made peace with your illness?	1	2	3	4
4. Do you feel well loved now?	1	2	3	4
5. To what extent do you feel a sense of inner calm and tranquility?	1	2	3	4
6. To what extent do changes in your physical appearance upset you?	1	2	3	4
7. To what extent does worry about your illness make it difficult for you to live day to day?	1	2	3	4
8. To what extent do you feel like it is unfair for you to have heart failure right now?	1	2	3	4
9. To what extent do you feel like your life, as you know it, is over?	1	2	3	4
10. To what extent do you feel angry about your illness?	1	2	3	4
11.To what extent do you feel that your illness has beaten you down?	1	2	3	4
12. To what extent do you feel ashamed of or embarrassed by your current condition?	1	2	3	4

© 2018 Allen LA et al. JAMA Internal Medicine.

Perceived Stress Scale

American Sociological Association, Cohen S, Kararch T, and Mermelstein R, 1983.

	Never	***Almost Never***	***Sometimes***	***Fairly Often***	***Very Often***
1. In the last month, how often have you been upset because of something that happened unexpectedly?	0	1	2	3	4
2. In the last month, how often have you felt that you were unable to control the important things in your life?	0	1	2	3	4
3. In the last month, how often have you felt nervous and "stressed"?	0	1	2	3	4
4. In the last month, how often have you felt confident about your ability to handle your personal problems?	0	1	2	3	4
5. In the last month, how often have you felt that things were going your way?	0	1	2	3	4
6. In the last month, how often have you found that you could not cope with all the things that you had to do?	0	1	2	3	4
7. In the last month, how often have you been able to control irritations in your life?	0	1	2	3	4
8. In the last month, how often have you felt that you were on top of things?	0	1	2	3	4
9. In the last month, how often have you been angered because of things that were outside of your control?	0	1	2	3	4
10. In the last month, how often have you felt difficulties were piling up so high that you could not overcome them?	0	1	2	3	4

© 2018 Allen LA et al. JAMA Internal Medicine.

The Patient Health Questionnaire-2 (PHQ-2)
Spitzer RL, Williams JBW, Kroenke K. Pfizer Inc.2003.

Use a circle or check mark to indicate your answer.

Over the <u>last two weeks</u>, how often have you been bothered by any of the following problems?

	Not at all	***Several days***	***More than half the days***	***Nearly every day***
1. Little interest or pleasure in doing things.	0	1	2	3
2. Feeling down, depressed, or hopeless.	0	1	2	3

© 2018 Allen LA et al. JAMA Internal Medicine.

EQ Visual Analogue Scale

1998 EuroQol Group EQ-5D™ is a trade mark of the EuroQol Group.

To help people say how good or bad a health state is, we have drawn a scale (rather like a thermometer) on which the best state you can imagine is marked 100 and the worst state you can imagine is marked 0.

We would like you to indicate on this scale how good or bad your own health is today, in your opinion. Please do this by drawing a line from the box below to whichever point on the scale indicates how good or bad your health state is today.

Your own health state today

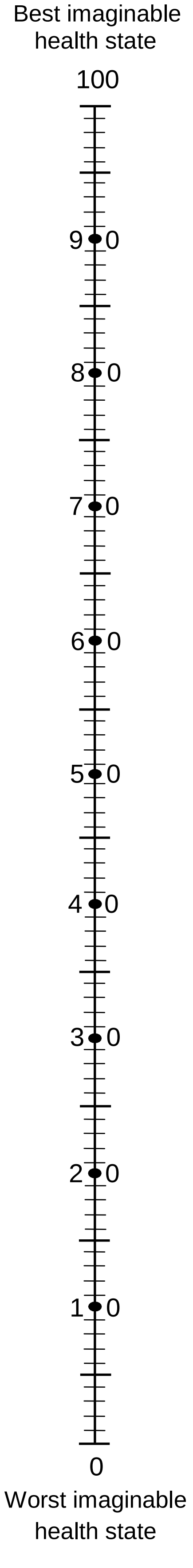

© 2018 Allen LA et al. JAMA Internal Medicine.

MISSING DATA

Methods: Missing data were characterized at each time point. For participants who were still alive at these time points, we used multiple imputation to impute missing values, repeated our primary analyses procedures, and combined results across imputation. For the knowledge analysis, covariates for the imputation included: study period, site, age, years since heart failure diagnosis, inpatient status, education, employment, LVAD eligibility, and LVAD decision. For the values-choice analysis, covariates also included: PEACE illness acceptance score, PHQ-2 score, and values scores. Results were similar to primary analysis results.

Results: Survey data were missing at baseline in 8.1% of control and 15.0% of intervention patients, 1-month in 23.0% of control and 30.1% of intervention patients, and 6-month in 27.4% of control and 40.7% of intervention patients. Missing data were most commonly due to death: 13 control and 8 intervention at 1 month, 14 control and 14 intervention at 6 months. Patients declining LVAD or who were found not to be a candidate for LVAD had higher rates of missing data at 1 month as compared to patients accepting LVAD. We did not observe any relationship between patient age, sex, race, education, income, or marital status with missing data, but did observe a difference with baseline data by site, study period, and enrollment location. Differences in those with complete versus missing data are available next within this supplement.

© 2018 Allen LA et al. JAMA Internal Medicine.

Participant Characteristics by Missing Primary Knowledge (at Baseline 1 and Baseline 2) and Values (at 1-Month) Outcomes

	N (%)					
	Knowledge Measure Availability (Baseline 1 and Baseline 2)			Values Measure Availability (1-Month)		
	Available (N=208)	Incomplete (N=40)	P-value	Available (N=179)	Incomplete (N=69)	P-value
**Age, mean (SD)**	63.23 (9.65)	64.00 (11.37)	0.69	62.85 (9.99)	64.67 (9.71)	0.19
**Sex**						
**Female**	31 (79.5%)	8 (20.5%)	0.42	24 (61.5%)	15 (38.5%)	0.11
**Male**	177 (84.7%)	32 (15.3%)		155 (74.2%)	54 (25.8%)	
**Race/Ethnicity**						
**White, non-Hispanic**	167 (88.8%)	21 (11.2%)	0.91	144 (76.6%)	44 (23.4%)	0.77
**Black**	27 (87.1%)	4 (12.9%)		22 (71.0%)	9 (29.0%)	
**Other**	12 (85.7%)	2 (14.3%)		11 (78.6%)	3 (21.4%)	
**Education**						
**High school or less**	76 (85.4%)	13 (14.6%)	0.24	68 (76.4%)	21 (23.6%)	0.95
**Some college or more**	132 (90.4%)	14 (9.6%)		111 (76.0%)	35 (24.0%)	
**On disability**	59 (86.8%)	9 (13.2%)	0.75	51 (75.0%)	17 (25.0%)	0.95
**Household income**						
**≤$40,000**	89 (88.1%)	12 (11.9%)	0.74	74 (73.3%)	27 (26.7%)	0.18
**>$40,000**	103 (89.6%)	12 (10.4%)		93 (80.9%)	22 (19.1%)	
**Married**	144 (88.3%)	19 (11.7%)	0.90	126 (77.3%)	37 (22.7%)	0.54
**First diagnosed with heart failure**						
**Within past 2 years**	22 (81.5%)	5 (18.5%)	0.43	18 (66.7%)	9 (33.3%)	0.25
**2-4 years**	24 (85.7%)	4 (14.3%)		22 (78.6%)	6 (21.4%)	
**4 or more years**	150 (89.3%)	18 (10.7%)		132 (78.6%)	36 (21.4%)	
**Unsure**	9 (100%)	0 (0.0%)		5 (55.6%)	4 (44.4%)	
**Did not answer**	3 (75.0%)	1 (25.0%)		2 (50.0%)	2 (50.0%)	
**Enrollment location**						
**Outpatient**	43 (74.1%)	15 (25.9%)	0.07	42 (72.4%)	16 (27.6%)	0.88
**Inpatient (non-ICU)**	114 (87.0%)	17 (13.0%)		93 (71.0%)	38 (29.0%)	
**ICU**	51 (86.4%)	8 (13.6%)		44 (74.6%)	15 (25.4%)	
**LVAD Treatment Outcome**						

© 2018 Allen LA et al. JAMA Internal Medicine.

**No LVAD**	57 (76.0%)	18 (24.0%)	<0.001	35 (46.7%)	40 (53.3%)	<0.001
**LVAD**	151 (92.1%)	13 (7.9%)		144 (87.8%)	20 (12.2%)	
**Study period**						
**Control**	121 (89.6%)	14 (10.4%)	0.007	103 (76.3%)	32 (23.7%)	0.11
**Intervention**	87 (77.0%)	26 (23.0%)		76 (67.3%)	37 (32.7%)	
**Site**						
**1**	36 (87.8%)	5 (12.2%)	<0.001	28 (68.3%)	13 (31.7%)	0.09
**2**	20 (90.9%)	2 (9.1%)		15 (68.2%)	7 (31.8%)	
**3**	33 (60.0%)	22 (40.0%)		34 (61.8%)	21 (38.2%)	
**4**	35 (97.2%)	1 (2.8%)		32 (88.9%)	4 (11.1%)	
**5**	31 (88.6%)	4 (11.4%)		24 (68.6%)	11 (31.4%)	
**6**	53 (89.8%)	6 (10.2%)		46 (78.0%)	13 (22.0%)	

© 2018 Allen LA et al. JAMA Internal Medicine.
